# Endocardium differentiation through *Sox17* expression in endocardium precursor cells regulates heart development in mice

**DOI:** 10.1038/s41598-019-48321-y

**Published:** 2019-08-16

**Authors:** Rie Saba, Keiko Kitajima, Lucille Rainbow, Silvia Engert, Mami Uemura, Hidekazu Ishida, Ioannis Kokkinopoulos, Yasunori Shintani, Shigeru Miyagawa, Yoshiakira Kanai, Masami Kanai-Azuma, Peter Koopman, Chikara Meno, John Kenny, Heiko Lickert, Yumiko Saga, Ken Suzuki, Yoshiki Sawa, Kenta Yashiro

**Affiliations:** 1 Centres for Microvascular Research and for Endocrinology, William Harvey Research Institute, Barts and The London School of Medicine and Dentistry, Queen Mary University of London, London, UK; 2 Cardiac Regeneration and Therapeutics, Graduate School of Medicine, Osaka University, Osaka, Japan; 3 Department of Developmental Biology, Graduate School of Medical Sciences, Kyushu University, Fukuoka, Japan; 4 Centre for Genomic Research, Institute of Integrative Biology, University of Liverpool, Liverpool, UK; 5 Institute of Diabetes and Regeneration Research, Helmholtz Zentrum München, Neuherberg, Germany; 6 Department of Veterinary Anatomy, The University of Tokyo, Tokyo, Japan; 7 Department of Experimental Animal Models for Human Disease, Graduate School of Medical and Dental Sciences, Tokyo Medical and Dental University, Tokyo, Japan; 8 Department of Pediatrics, Graduate School of Medicine, Osaka University, Osaka, Japan; 9 Department of Biophysics and Biochemistry, Graduate School of Medicine, Osaka University, Osaka, Japan; 10 Department of Cardiovascular Surgery, Graduate School of Medicine, Osaka University, Osaka, Japan; 11 Division of Molecular Genetics and Development, Institute for Molecular Bioscience, The University of Queensland, Brisbane, Australia; 12 Teagasc Food Research Centre, Moorepark, Co Cork Ireland; 13 Division of Mammalian Development, Genetic Strains Research Center, National Institute of Genetics, Shizuoka, Japan; 14 Division of Anatomy and Developmental Biology, Department of Anatomy, Kyoto Prefectural University of Medicine, Kyoto, Japan

**Keywords:** Development, Differentiation

## Abstract

The endocardium is the endothelial component of the vertebrate heart and plays a key role in heart development. Where, when, and how the endocardium segregates during embryogenesis have remained largely unknown, however. We now show that *Nkx2-5*^+^ cardiac progenitor cells (CPCs) that express the Sry-type HMG box gene *Sox17* from embryonic day (E) 7.5 to E8.5 specifically differentiate into the endocardium in mouse embryos. Although *Sox17* is not essential or sufficient for endocardium fate, it can bias the fate of CPCs toward the endocardium. On the other hand, *Sox17* expression in the endocardium is required for heart development. Deletion of *Sox17* specifically in the mesoderm markedly impaired endocardium development with regard to cell proliferation and behavior. The proliferation of cardiomyocytes, ventricular trabeculation, and myocardium thickening were also impaired in a non-cell-autonomous manner in the *Sox17* mutant, likely as a consequence of down-regulation of NOTCH signaling. An unknown signal, regulated by *Sox17* and required for nurturing of the myocardium, is responsible for the reduction in NOTCH-related genes in the mutant embryos. Our results thus provide insight into differentiation of the endocardium and its role in heart development.

## Introduction

Heart development is one of the earliest events of vertebrate organogenesis. Cardiac progenitor cells (CPCs) give rise to the myocardium, endocardium, epicardium, smooth muscle, fibroblasts, and endothelium of coronary vessels in the mammalian heart. In the mouse embryo, CPCs originate between embryonic day (E) 6.25 and E7.5 from nascent mesoderm cells in the primitive streak that express the basic helix-loop-helix (bHLH) transcription factor gene *Mesp1*. These mesoderm cells are a part of the lateral plate mesoderm (LPM) and populate the heart field as CPCs at the most anterior region of the embryo, and they begin to express CPC markers at the early allantoic bud (EB) stage (E7.5). In addition to well-validated CPC marker genes encoding transcription factors—including *Nkx2-5*, *Isl1*, and *Tbx5*^1–3^—analysis of alternative markers has provided the basis for a model of the cell lineage tree and the mechanics of cardiomyocyte differentiation^4,5^. Among the cell types constituting the heart, the development of endocardial cells has remained largely uncharacterized.

The endocardium is the innermost layer and endothelial component of the heart. During heart development, the endocardium provides a source of cells for the valves, the membranous portion of the ventricular septum, the endothelium of coronary vessels, and cardiac fibroblasts. It plays a key role in ventricular trabeculation, myocardial compaction, as well as valve and coronary vessel formation. In the mouse embryo, endocardial cells are first recognized morphologically as a proendocardium layer between the myocardium and definitive endoderm layers at the one- to two-somite stage, when expression of the transcription factor gene *Nfatc1*, an early and unique marker of the endocardium, is initiated^6–8^.

In mice, *Mesp1*-expressing nascent mesoderm cells in the primitive streak have been shown to include two types of cell that give rise to the endocardium: unipotent endocardium precursors, and bipotent cardiac progenitors that contribute to cardiomyocytes and the endocardium^9^. No molecular marker has been identified to distinguish these two cell types from each other, but both likely become *Nkx2-5*-positive CPCs^1,10^. Downstream of *Mesp1*, the ETS-related transcription factor gene *Etv2* (also known as *Er71* or *Etsrp71*) plays a role in endocardium development at the top of the endothelial genetic cascade^11^. Disruption of *Etv2* was found to result in loss of the endocardium^12,13^, suggesting that the endocardium originates from *Etv2*^+^ mesoderm. It remains unclear, however, whether *Etv2* determines endocardium fate or only confers competence for endocardium differentiation. Downstream of *Etv2*, a network of transcription factors—including ETS, SOX, GATA, and RBPJκ—regulates endocardium differentiation. These factors likely activate expression of *Kdr/Flk1* and *Dll4* in the endocardium^14,15^, with these genes being implicated in vascular endothelial growth factor (VEGF) and NOTCH signaling essential for endocardium development^16,17^. However, the network of transcription factors responsible for the induction of endocardium fate remains largely unidentified.

Among three Sry-type HMG box F (SOXF) transcription factor genes—*Sox7*, *Sox17*, and *Sox18*—expression of *Sox17* was long regarded as specific for the endoderm including the visceral and definitive endoderm^18^. However, *Sox17* was subsequently shown to be essential for vascular development and definitive haematopoiesis^19,20^. Lineage tracing for *Sox17*-expressing cells revealed that they contribute not only to the endoderm but also to the mesoderm that gives rise to the endocardium^21^. Conditional knockout of *Sox17* in the endothelial lineage of mice resulted in the loss of vascular rearrangement in the embryo and yolk sac as well as in that of definitive haematopoiesis^19,20^. With regard to cardiac development, differentiation of mouse embryonic stem cells into cardiomyocytes *in vitro* was suppressed in a non-cell-autonomous manner by *Sox17* ablation, although the identity of *Sox17*-expressing cells that are required for cardiomyogenesis has been unclear^22^.

Here we show that *Sox17* is expressed in a subset of CPCs during the early phase of mouse cardiac development. Single-cell gene expression profiling revealed that ~20% to 30% of *Nkx2-5*^+^ CPCs are positive for *Sox17* expression. SOX17^+^ mesoderm cells were found in the region corresponding to the heart field in E7.5 mouse embryos. This *Sox17* expression is transient, persisting up to E8.5 at the latest. Tracing of *Sox17*^+^ cells showed that the endocardium originates from *Sox17*^+^ mesoderm cells. Gain-of-function and mesoderm-specific loss-of-function analyses for *Sox17* in mouse embryos revealed that *Sox17* is required for heart development, although it is not essential or sufficient for endocardial fate. Single-cell gene expression profiling for the mesoderm-specific *Sox17* mutant showed that disruption of *Sox17* resulted in misregulation of the transcriptome in endocardial and myocardial cells in a cell-autonomous and non-cell-autonomous manner, respectively. Our findings thus provide insight into development of the endocardium and its relation to heart morphogenesis.

## Results

### *Sox17* expression in CPCs

**Figure 1:**
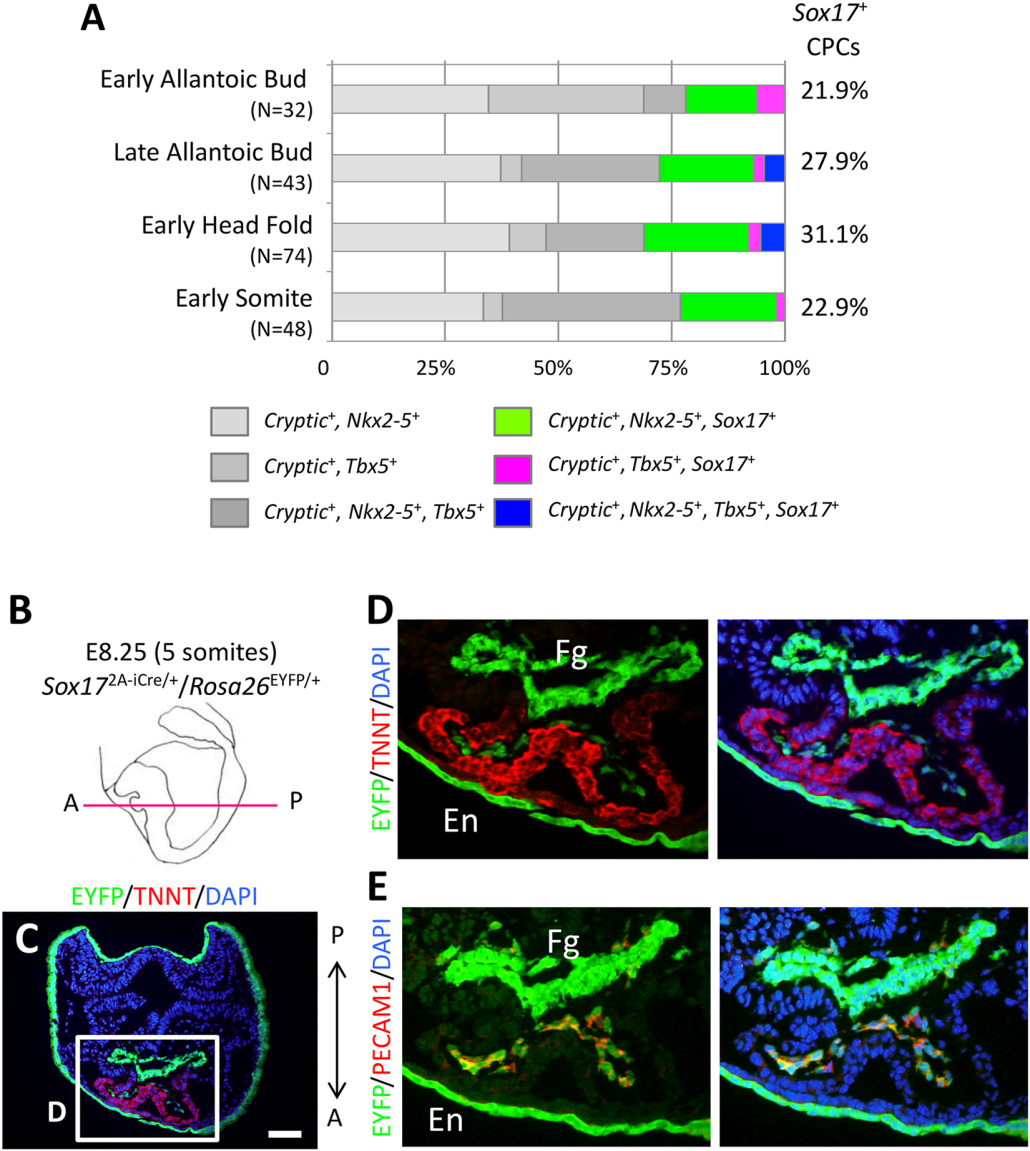
*Sox17*^+^ CPCs specific for the endocardium differentiation. (**A**) Proportion of *Sox17*-expressing CPCs (*Cryptic*^+^, *Nkx2-5*^+^ and/or *Tbx5*^+^ cells) in mouse embryos at E7.5 (early allantoic bud, late allantoic bud, and early head fold stages) and E8.5 (early somite stage). *N* values indicate the number of cells examined. The data are derived from our previous study^23^. (**B**) Schematic representation of a mouse embryo at the five-somite stage (E8.25) as a left lateral view. The magenta line shows the sectional plane along the anterior (**A**)-posterior (P) axis in (**C**). (**C**–**E**) Immunofluorescence micrographs for EYFP (green), TNNT (red in **C**,**D**) and PECAM1 (red in **E**). Nuclei (blue) were stained with 4′,6-diamidino-2-phenylindole (DAPI). The boxed region in *C* is shown at higher magnification in *D*. The section shown in *E* is adjacent to that in *D*. Fg, foregut; En, endoderm. Scale bar, 100 µm.

We previously performed single-cell gene expression profiling of mouse embryonic CPCs from the EB to early head fold (EHF) stage^23,24^. We validated cell types based on the expression of marker genes including *Sox2* for the epiblast or neural ectoderm, *Sox17* for the endoderm or arterial endothelial cells^18,25^, *Cryptic* (also known as *Cfc1*) for LPM, and *Nkx2-5* or *Tbx5* for CPCs^1,3^ as described previously^23^. These data revealed that *Sox17* was expressed in 21.9% of CPCs (*Cryptic*^+^, *Nkx2-5*^+^ and/or *Tbx5*^+^) at the EB stage (Fig. 1A). We also confirmed that ~20% to 30% of CPCs continued to express *Sox17* up to the early somite stage (Fig. 1A), suggesting that *Sox17* plays a role in a subset of CPCs.

We next examined the distribution of SOX17-expressing CPCs in mouse embryos by immunofluorescence analysis (Fig. S1). At the EHF stage, SOX17^+^ CPCs were marked simultaneously by the CPC marker NKX2-5 in the most anterior portion of the embryo corresponding to the heart field (Fig. S1). To distinguish LPM cells from the endoderm, we studied *Mesp1*^Cre/+^/*Rosa26*^EYFP/+^ mouse embryos, in which *Mesp1*-expressing mesoderm cells are labeled with enhanced yellow fluorescent protein (EYFP)^26,27^. Whereas SOX17^+^ cells were rarely detected among the EYFP^+^ LPM cells in embryos at the EB and late allantoic bud stages, SOX17^+^/EYFP^+^ cells were readily apparent at the EHF stage (Fig. S2). As the endoderm layer thickened and the shape of the foregut pocket became more obvious from the late head fold to zero-somite stage (E8.0), SOX17^+^ LPM cells became localized more exclusively to a region near the dorsal side of the foregut pocket fold (Fig. S3A). From the three-somite stage (E8.25), the number of SOX17^+^ LPM cells decreased concomitantly with the decline in expression of SOX17 in the anterior definitive endoderm (Fig. S3B). Only a few SOX17^+^ LPM cells were apparent at the sinus venosus. These results thus showed that SOX17 is expressed in CPCs during early embryogenesis.

### Contribution of *Sox17*^+^ CPCs to the endocardium

We next characterized *Nkx2-5*^+^/*Sox17*^+^ CPCs by polymerase chain reaction (PCR) analysis of marker gene expression with single-cell cDNA. Expression of the endothelial marker genes *Dll4* and *Pecam1* was highly correlated with that of *Sox17* in *Nkx2-5*^+^ CPCs at the early somite stage (Fig. S4A), whereas the expression of other marker genes, such as *Actn2* (cardiomyocytes) or *Acta2* (smooth muscle cells), was not (Fig. S5A)^28–31^. These results suggested that *Sox17*-expressing cells contribute to endothelial-like cells, a conclusion also supported by the preferential expression of *Tal1*, *Kdr*, *Etv2* and *Notch1*, marker genes for arterial endothelial precursor cells, in *Nkx2-5*^+^/*Sox17*^+^ CPCs at the early somite stage (>75% for *Tal1*, *Etv2* and *Notch1*) as well as in *Nkx2-5*^+^ CPCs at the EB stage (100% and 72.7% for *Kdr* and *Etv2*, and for *Notch1*, respectively) (Fig. S4B,C)^12,13,32,33^. An endocardial marker gene, *Nfatc1*, also showed a similar expression pattern to *Tal1* in *Nkx2-5*^+^/*Sox17*^+^ CPCs (Fig. S4B). Given that the endothelial component originating from *Nkx2-5*-expressing CPCs becomes the endocardium, it is likely that *Sox17*-expressing CPCs give rise to the endocardium^34^. Of note, other *SoxF* genes, *Sox7* and *Sox18*, also showed highly correlated with that of *Sox17* in *Nkx2-5*^+^ CPCs at the early somite stage (>75% for both of *Sox7* and *Sox18*) (Fig. S5B), showing the redundant expression patterns among *SoxF* genes in the endocardium lineage.

To examine further whether endocardium cells are indeed derived from *Sox17*-expressing mesoderm cells, we traced the lineage of *Sox17*-expressing cells in *Sox17*^Sox17-2A-iCre/+^/*Rosa26*^EYFP/+^ embryos (Fig. 1B–E)^21^, in which *Sox17*-expressing cells are labeled with EYFP. We found that the progeny of *Sox17*-expressing cells were all the PECAM1^+^ endocardium cells that were enclosed by the troponin T (TNNT)^+^ myocardium layer at the five-somite stage (E8.5), consistent with the results of a previous study^21^. *Sox17*^+^ CPCs are thus the specific precursors of the endocardium.

### *Sox17* expression biases CPCs to express an endothelial gene program

**Figure 2:**
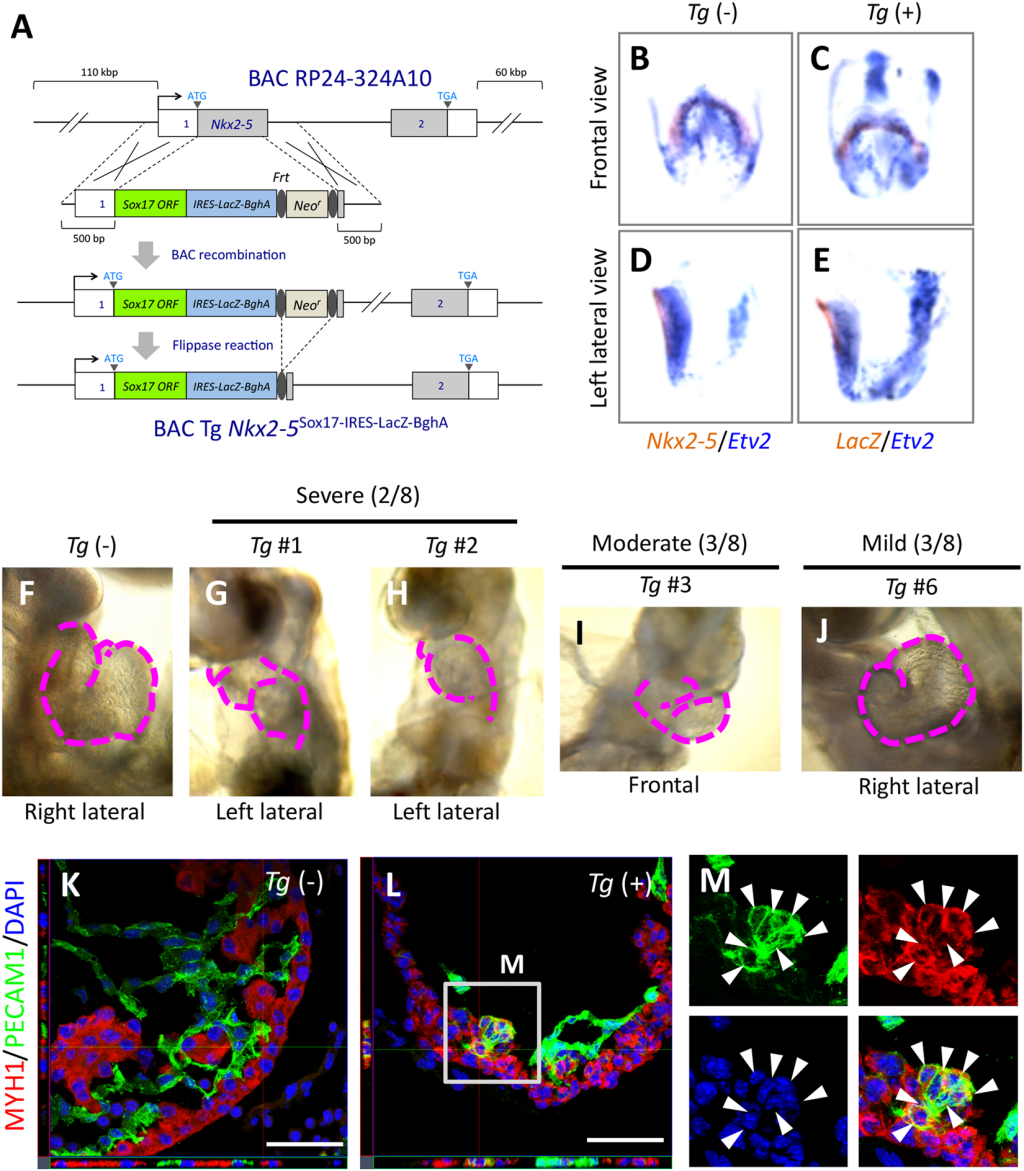
Gain of function of *Sox17* in *Nkx2-5*-expressing CPCs. (**A**) Construction of the BAC-based *Nkx2-5*^Sox17-IRES-LacZ^ *Tg*. (**B**–**E**) Double whole-mount *in situ* hybridization for *Etv2* (blue) and for either *Nkx2-5* (brown in **B**,**D**) or *LacZ* (brown in **C**,**E**) in E8.25 *Tg*(−) or *Tg*(+) embryos, respectively. (**F**–**J**) The heart of a *Tg*(−) embryo (**F**) and of *Tg*(+) embryos with severe (**G**,**H**), moderate (**I**), or mild (**J**) phenotypes at E9.5. Dashed magenta lines show the outline of the heart tube. (**K**–**M**) Immunofluorescence micrographs for the sarcomere myosin heavy chain MYH1 (red) and PECAM1 (green) in the left ventricle of the heart of E9.5 *Tg*(−) (**K**) and *Tg*(+) (**L**) embryos. (**M**) The boxed region in *L* is shown at higher magnification. Blue, DAPI; Arrowheads, cardiomyocytes expressing PECAM1; Scale bars, 100 µm.

The specificity of *Sox17* expression for the endothelial cell lineage suggested that *Sox17* plays a role in endocardial differentiation of CPCs. We therefore next examined whether *Sox17* expression is sufficient to establish endocardium cell fate in CPCs by forced expression of *Sox17* in *Nkx2-5*-expressing CPCs with the use of a bacterial artificial chromosome (BAC)-based *Nkx2-5*^Sox17-IRES-LacZ^ transgene (*Tg*) (Fig. 2A). Expression of the *Tg* was confirmed to mimic endogenous *Nkx2-5* expression in CPCs (Fig. 2B–E). The most severe defect of the *Tg*^+^ embryos at E9.5 was anomalous looping and structure of the heart tube (Fig. 2F–J), but no obvious morphological abnormalities were apparent before this stage. Inside the heart of both severely (Fig. 2K–M) and moderately (Fig. S6A,B) affected embryos, ventricular trabeculation was impaired. Apoptosis was frequently observed among MYH (a sarcomeric myosin heavy chain protein)-expressing cardiomyocytes in *Tg*^+^ heart tube (Fig. S6C–E), whereas the proliferation of NKX2-5^+^ cells inside the heart of *Tg*^+^ embryos was not significantly affected (Fig. S6F–H). The overall gain-of-function phenotype thus suggested that ectopic and excess *Sox17* expression is toxic. Of note, cardiomyocytes expressing PECAM1 were apparent in some *Tg*^+^ embryos with a severe phenotype, a phenomenon never observed in wild-type (WT) embryos (Fig. 2K–M), suggesting that *Sox17* expression biases the fate of CPCs toward an endothelial-like phenotype but is not sufficient to induce an endocardial cell fate in these cells. This conclusion is consistent with the observation that expression of *Etv2* was not detected in the *Tg*-expressing (*LacZ*-positive) area of *Tg*^+^ embryos (Fig. 2C,E). On the other hand, *Tg*^+^ embryos with a mild to moderate phenotype manifested pronounced aggregation of Isolectin B4 (an endothelial marker)-positive endocardium cells rather than a monolayer (Fig. S6B). The intimate interaction between the endocardium and myocardium was also missing. These findings suggested that *Sox17* expression in the endocardium must be maintained at an appropriate level for proper regulation of cell behavior.

### Cardiac defects induced by *Sox17* deletion in mesoderm

**Figure 3:**
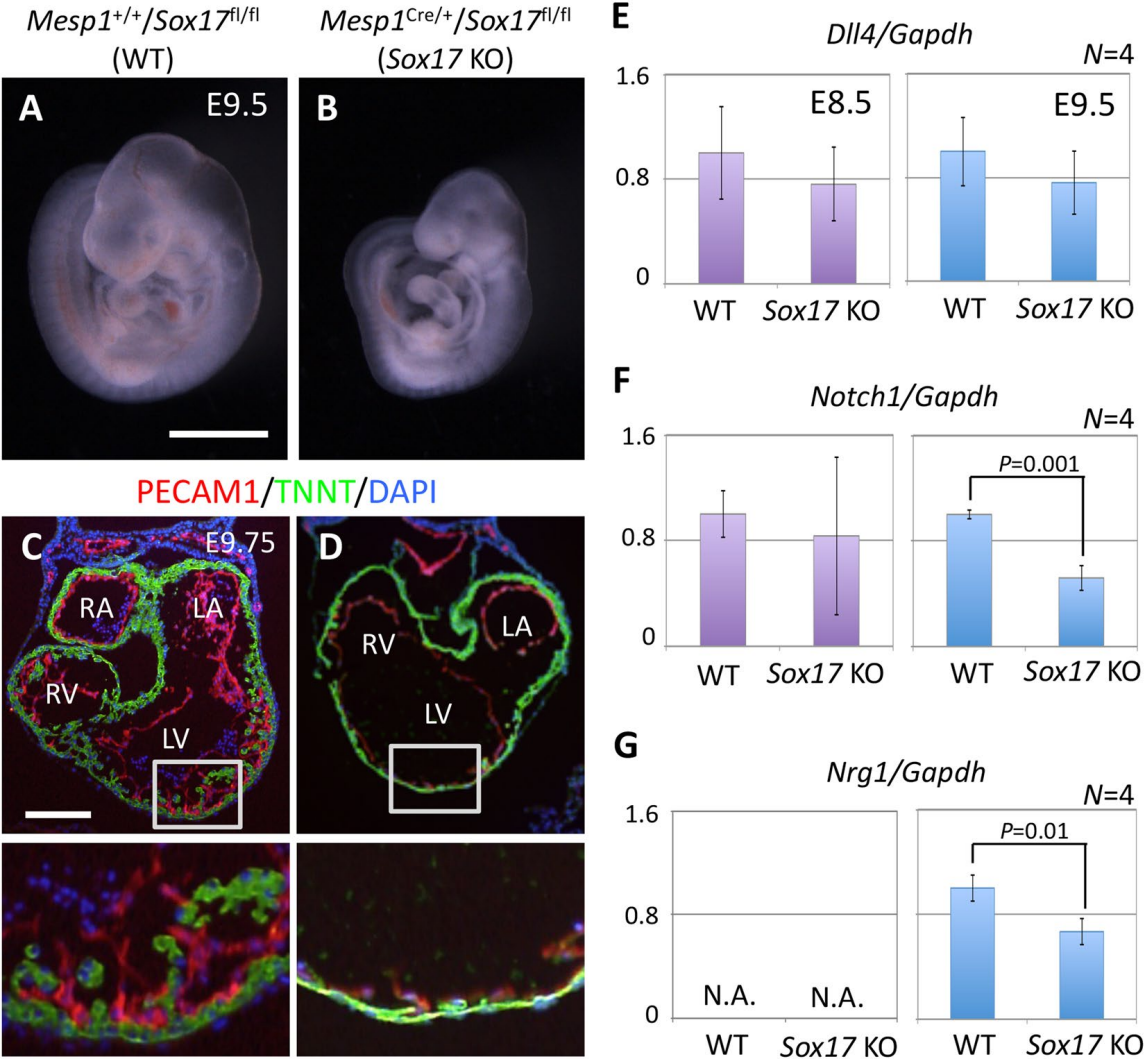
Cardiac defects associated with mesoderm-specific loss of function for *Sox17* in mouse embryos. (**A**,**B**) *Mesp1*^+/+^/*Sox17*^fl/fl^ (WT) (**A**) and *Mesp1*^Cre/+^/*Sox17*^fl/fl^ (*Sox17* KO) (**B**) embryos at E9.5. Scale bar, 1 mm. (**C**,**D**) Immunofluorescence micrographs in the heart of WT (**C**) and *Sox17* KO (**D**) embryos at E9.75. The boxed regions in the upper panels are shown at higher magnification in the lower panels. Red, PECAM1; Green, TNNT; Blue, DAPI; LA, left atrium; RA, right atrium; LV, left ventricle; RV, right ventricle. Scale bar, 100 µm. (**E**–**G**) Reverse transcription and real-time PCR analysis of the relative expression levels for the NOTCH signaling-related genes *Dll4* (**E**), *Notch1* (**F**), and *Nrg1* (**G**) in the heart of WT and *Sox17* KO embryos at E8.5 (left panels) and E9.5 (right panels). *Nrg1* expression at E8.5 was below the threshold for detection (N.A., not amplified). Only significant *P* values (Student’s *t* test) are indicated. Means ± SD.

To elucidate the physiological function of *Sox17* in endocardium development, we conducted conditional depletion of *Sox17*. Since it cannot be excluded that *Sox17* expression in CPCs starts earlier than *Nkx2-5*, we used the mice with *Mesp1*^Cre/+^ and floxed *Sox17* (*Sox17*^fl/fl^)^19^ alleles to knockout *Sox17* in all cardiac cell lineages. Although multiple anomalies were apparent in *Mesp1*^Cre/+^/*Sox17*^fl/fl^ embryos at E9.5, no obvious morphological abnormalities were detected before this stage. Growth retardation and ballooning of the pericardial sac were observed after approximately the 20-somite stage, suggestive of a severe defect in the peripheral circulation that likely resulted from embryonic heart failure (Fig. 3A,B). Since the defect of the primitive haematopoiesis is possible to cause cardiac malformation, we also confirmed the yolk sac development in the mutants (Fig. S7). The primitive haematopoiesis seemed not affected in the mutant at E9.5, consistent with the previous observation that a haematopoiesis defect was only found after E11.5 in *Sox17* mutant^19^. However, remodeling of the yolk sac vessels was also impaired in the mutant embryos. In WT embryos, the network of capillary vessels that connect to the blood islands, where primitive haematopoiesis gives rise to the production of primary erythrocytes, undergoes remodeling to form the larger vessel network of the yolk sac at E9.5 (Fig. S7). In contrast, such vascular rearrangement did not occur in the mutant. These results thus indicated that *Sox17* is essential for vascular development in addition to the heart development.

Examination of the heart tube revealed anomalous looping in *Mesp1*^Cre/+^/*Sox17*^fl/fl^ embryos at E9.5 (Fig. S8A–F). In WT embryos, the borders of the outflow tract, ventricle, and atrium were identified by folds with a sharp angle. However, the fold angles were obtuse in the mutant. The endothelial-like phenotype of the endocardium was not impaired in the heart of mutant embryos at E9.75, as revealed by the expression of PECAM1 (Fig. 3C,D), indicating that *Sox17* is not essential for endocardium fate in CPCs. However, the number of endocardium cells appeared to be reduced in the mutant. Whereas the endocardium was in intimate contact with the myocardium layer in WT embryos, such “touchdown” sites^35,36^ were far fewer in the mutant (asterisks in Fig. S9A–C). The size of cardiomyocytes was also reduced in the mutant embryos. Importantly, myocardial trabeculation was severely affected and the number of ventricular trabeculae composed of more than three cardiomyocytes was significantly reduced in the mutant (Fig. S9D). Together, these observations indicated that the physiological function of the endocardium was so anomalous in the *Sox17* mutant that maturation of the myocardium was affected in a non-cell-autonomous manner.

We next examined the expression levels of NOTCH signaling-related genes in the developing heart, given that a *Dll4-Notch1-Nrg1* pathway plays an important role in the differentiating endocardium as well as in ventricular trabeculation^37^. The expression level of *Dll4* did not differ significantly between WT and *Mesp1*^Cre/+^/*Sox17*^fl/fl^ embryos (Fig. 3E). At E8.5, the abundance of *Notch1* mRNA (Fig. 3F) and NOTCH1 protein (Fig. S8G,H) was unchanged in the mutant. However, at E9.5, the expression of *Notch1* and *Nrg1* (a downstream target of the NOTCH signal) in the heart was down-regulated significantly in the mutant embryos (Fig. 3F,G), suggesting that SOX17 does not directly induce NOTCH signaling but instead maintains it in the embryonic heart. Further, the expression levels of *Hey1* and *Hey2*, which regulate the cell proliferation of cardiomyocytes downstream of NOTCH signaling^38,39^, tended to be reduced in the mutant heart at E9.5 (Fig. S9E,F). The down-regulation of NOTCH signaling apparent at E9.5 is consistent with the previous finding that such signaling is required for trabeculation^40^.

### Transcriptome changes in differentiating cardiac cells induced by *Sox17* deletion

**Figure 4:**
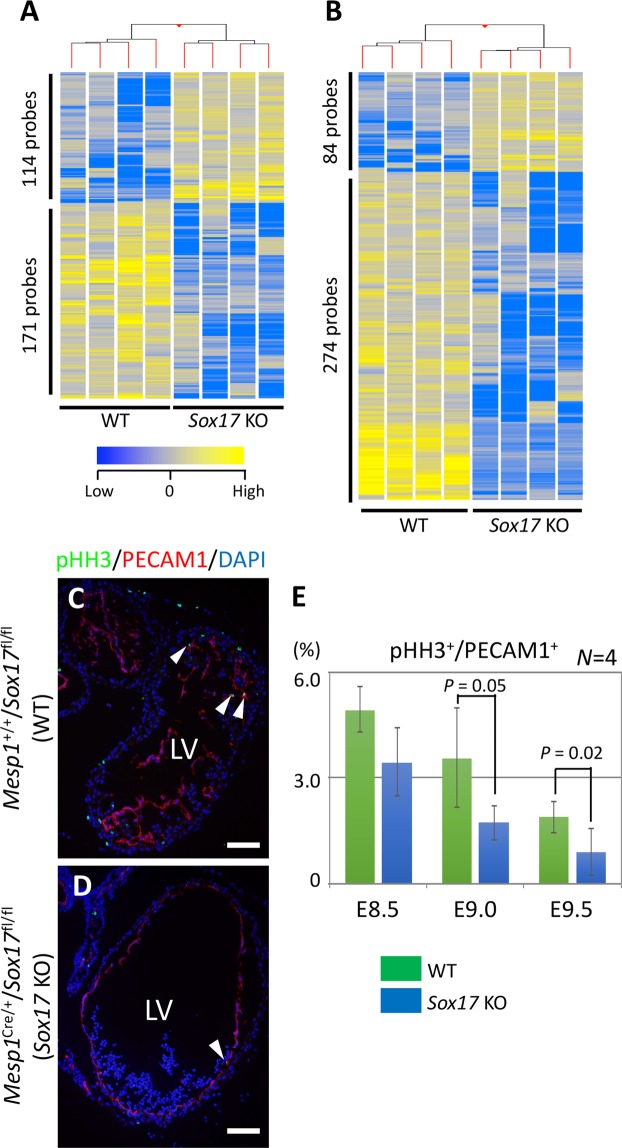
Altered gene expression profiles in the endocardium and myocardial cells of mesoderm-specific *Sox17* mutant embryos. (**A**,**B**) Hierarchical clustering and heat map for 285 (**A)** and 358 (**B**) probe sets for differentially expressed genes in the endocardium and myocardial cells, respectively, of WT and *Sox17* KO embryos at E8.5. (**C**,**D**) Immunofluorescence micrographs for pHH3 (green) and PECAM1 (red) in the heart of WT (**C**) and KO (**D**) embryos at E9.5. Blue is DAPI. Arrowheads indicate pHH3^+^ endocardium cells. LV, left ventricle; Scale bars, 50 µm. (**E**) Proportion of pHH3^+^ cells among PECAM1^+^ cells in the heart of WT and *Sox17* KO embryos from E8.5 to E9.5. Means ± SD. Only *P* values of ≤0.05 (Student’s *t* test) are indicated.

The heart anomalies of mesoderm-specific *Sox17* mutant embryos were not apparent until E9.5, even though SOX17 is expected to function from E7.5 to E8.5 (Figs 1; S1–S3). We therefore examined whether the loss of function for *Sox17* affects the endocardium from the earliest phase of its development, before the morphological abnormality is evident, with the use of single-cell microarray-based expression profiling of endocardium cells at E8.5 (nine-somite stage). Marked differences in gene expression profiles were detected between WT and *Mesp1*^Cre/+^/*Sox17*^fl/fl^ cells. The normalized signal intensities of 114 and 171 probe sets (2.71% of total probe sets called “present”) were increased and decreased, respectively, by a factor of at least 5 in endocardium cells of the mutant relative to those of WT embryos (Fig. 4A; Table S1; SI, Gene Lists 1, 2). The gene expression profile of cardiomyocytes of E8.5 mutant embryos also differed from that of control cardiomyocytes, with the normalized signal intensities of 84 and 274 probe sets (4.78% of total probe sets called “present”) being increased and decreased, respectively (Fig. 4B; Table S1; SI, Gene Lists 3, 4), supporting the notion that *Sox17* expression in the endocardium contributes to regulation of the genetic program for cardiomyocyte differentiation from the early stage of heart development. Of note, mitochondria related genes (*Acsl4*, *Mcat*, *Micu2*, *Mrpl4*, *Mrpl9*, *Mrpl4*, *Mrpl13*, *Mrpl30*, *Mrpl36*, *Mrpl38*, *Mrps24*, *Ndufa4*, *Ndufa10*, *Tomm34*, *Oxa1l*, *Slc25a13*), sarcomere-related genes (*Hrc*, *Speg*, *Tpm4*) and cardiac development-related genes (*Cited2*, *Gata4*, *Irx4*, *Ldb3*, *Pfkm*, *Tln1*) were significantly down-regulated in the mutant cardiomyocytes, suggesting that cardiomyocyte maturation was impaired (Gene List 4).

Pathway analysis for the lists of genes whose expression was affected in mutant embryos revealed that molecules related to “cellular growth and proliferation,” “cellular development,” and “cell cycle” were significantly enriched for both endocardium cells and cardiomyocytes (Tables S2, S3). Examination of cell proliferation in the heart from E8.5 to E9.5 revealed that the ratio of KI67^+^ (mitotic) endocardium cells or cardiomyocytes did not differ significantly between WT and mutant embryos at E8.5 or E9.0 but was significantly reduced in the mutant at E9.5 (Fig. S10A,B). Similarly, the ratio of phosphorylated histone H3 (pHH3)-positive (M-phase) cells in the endocardium of mutant embryos was significantly reduced at E9.0 (Fig. 4C–E). A significant reduction in the ratio of cardiomyocytes in M phase was apparent in the mutant at E9.5 (Fig. S10C–E), consistent with the notion that an initial anomalous molecular event in the endocardium subsequently affects the myocardium. Given that a reduction in *Notch1* expression in the mutant heart was not apparent until E9.5 (Fig. 3F), a primary event caused by the lack of *Sox17* expression in the endocardium might secondarily induce down-regulation of *Notch1* expression and thereby affect cell proliferation and trabeculation in the myocardium. Together, these results suggest that *Sox17* expression in the endocardium precursor cells is essential for regulation of endocardium development in a cell-autonomous manner and for that of myocardium development in a non-cell-autonomous manner.

## Discussion

We have here shown that *Sox17* is transiently expressed from E7.5 to E8.5 specifically in CPCs undergoing differentiation into the endocardium in mouse embryos. Gain-of-function and loss-of-function analyses revealed that *Sox17* is neither necessary nor sufficient for the induction of endocardial cell fate in CPCs. However, its expression in endocardium precursor cells is required for proper heart development and biases CPCs toward an endothelium-like phenotype. Our results suggest that SOX17 renders endocardium cells competent for proliferation and interaction with cardiomyocytes, with such interaction regulating cardiomyocyte growth and maturation (Fig. S10F).

Much of the available information relevant to endocardium differentiation has been derived from studies of the development of haematopoietic stem cells and the arterial endothelium. In mice, *Etv2* is essential for establishment of the endothelial and haematopoietic cell lineages in *Mesp1*^+^ mesoderm, serving as a downstream target of bone morphogenetic protein (BMP), NOTCH, and WNT signaling pathways^20^. Null mutation of *Etv2* results in embryonic mortality and loss of endothelial cells including the endocardium as well as of blood cells^12,13^. In addition, *Notch1*, which plays an important role in development of haemogenic arterial endothelial cells, is also required for development of the endocardium^41–43^. Mesodermal cells expressing *Notch1* at E6.5 were found to contribute mostly to the endocardium^44^. Given that *Sox17* appears to function in the haematopoietic lineage and that the endocardium likely possesses haemogenic ability^19,45^, the endocardium may share a common or similar developmental gene program including *Sox17* with the haematopoietic lineage. This notion is further supported by the observation that zebrafish mutants of *cloche*, which encodes a bHLH-PAS-type transcription factor and is expressed in endothelial and haematopoietic precursors, do not develop an endocardium or haematopoietic stem cells^46,47^. Of interest, NKX2-5 was shown to transactivate *Etv2* in *Nkx2-5*^+^ cells that contribute to the endocardium, suggesting that the cardiac program functions upstream of the endothelial-haemogenic progenitor gene program essential for endocardium development^48^. However, the endothelial-haemogenic program including *Etv2* and *Tal1* prevents the induction of cardiomyocyte fate in CPCs^48,49^. Endocardium fate determination might thus be initiated by the cardiac program coupled to the endothelial-haemogenic program among *Mesp1*-expressing nascent mesoderm cells, with the endothelial-haemogenic program subsequently suppressing the cardiomyocyte program after commitment to the endocardium. Validation of the mechanism underlying fate determination for the endocardium will require further studies of the roles of *Mesp1*, *Sox17*, *Etv2*, *Notch1*, and other early endothelial-haemogenic factor genes.

*Sox17* has been shown to be required for vascular development. Its expression was thus found to promote sprouting angiogenesis during retinal vascularization and tumor angiogenesis^20,50^. Loss or gain of function of *Sox17* in the endothelial lineage of mouse embryos resulted in decreased and increased vascular density, respectively^51^. SOX17 was suggested to destabilize vascular endothelial cells and thereby to secure the motility of tip cells during angiogenesis by regulating the proliferation, adhesion, and cytoskeletal organization of endothelial cells as well as the extracellular matrix. The phenotypes resulting from loss or gain of function of *Sox17* in our study are likely consistent with such a role, given the observed defects in cell shape, cell proliferation, and cell-cell interaction (Figs 2–4, Figs S6–10). Endocardial cells that sprout toward the myocardium via an angiogenesis-like process may thus generate the touchdown sites at which cardiomyocytes are able to initiate their trabeculation^35,36^. For the endocardium to exert such an action, *Sox17* must be expressed at an appropriate level, given the phenotypes associated with its gain or loss of function (Figs 2, 3). On the other hand, a specific role for *Sox17* in heart development is difficult to discern because of the functional redundancy of SOXF transcription factor genes. *Sox7* is expressed in a subset of the *Mesp1*^+^ cell lineage from the earliest phase^44^, and *Sox7* mutant embryos manifest an overall phenotype similar to that of embryos lacking *Sox17*^52^. Identification of the direct targets of SOX17 and the other SOXF transcription factors should provide insight into the roles of these proteins in development.

Activated DLL4-NOTCH1 signaling in the endocardium was shown to be essential for ventricular trabeculation, with the consequent up-regulation of *Ephrin B2-EphB4-Nrg1* signaling promoting the differentiation of cardiomyocytes^36,37^. We found that loss of function of *Sox17* resulted in defective trabeculation, likely as a result of the significant down-regulation of *Notch1* and *Nrg1* expression in the heart apparent at E9.5. These findings appear consistent with the previous observation that SOX17 directly activates *Notch1* to establish the haemogenic endothelium^42^. However, expression of *Notch1* was not affected in our *Sox17* loss-of-function mutant at E8.5, indicating that *Sox17* is dispensable for the induction of *Notch1* expression at the earliest phase of endocardium development, in contrast to the situation for haematopoietic stem cell development. The reduction in the level of *Notch1* expression in our *Sox17* mutant at E9.5 might be a secondary impairment due to a primary defect in the endocardium, or *Sox17* may be required for maintenance of *Notch1* expression. Alternatively, rather than SOX17, one or both of the other SOXF transcription factors (SOX7, SOX18) might directly activate *Notch1* in the endocardium lineage. Further studies of the relative roles of SOX17 and the other SOXF proteins should provide insight into the development of the endocardium.

## Methods

### Mice

*Sox17*^fl^ (MGI ID: 3717121)^19^ and *Rosa26*^EYFP^ Cre reporter (MGI ID: 2449038)^27^ mice were obtained from The Jackson Laboratory. *Mesp1*^Cre^ mice (MGI ID: 2176467) were described previously^26^. All animal procedures were performed under project licenses (70/7254 and 70/7449) approved by the Home Office according to the Animals (Scientific Procedures) Act 1986 in the U.K., or with approval of the Osaka University Animal Experimentation Committee (license number: 29-039-004) in Japan.

### Staging of mouse embryos

Developmental stages of mouse embryos were classified according to morphology as previously described^23^. The morning of the day of vaginal plug detection was set as E0.5.

### BAC transgenesis

The construction of BAC transgene and transgenic mice production were performed as previously described^23,53^. The BAC *Nkx2-5*^Sox17-IRES-LacZ-BghpA^ *Tg* was constructed with a BAC recombination system as shown in Fig. 2A. The BAC clone RP24-324A10 contains 175.0 kb of the mouse *Nkx2-5* locus and drives expression of the *Sox17*-*IRES-LacZ* cassette (containing full-length mouse *Sox17* cDNA) according to the genomic context of *Nkx2-5*. A *Sox17*-*IRES-LacZ-BghpA* cassette was introduced in-frame into the mouse *Nkx2-5* gene of BAC clone RP24-324A10. For recombination with the BAC, left-arm XbaI-XhoI and right-arm EcoRI-EcoRV fragments were amplified by PCR independently with the primer sets *Nkx2-5*-L-arm-F (5′-XbaI site-GTCGACCGTTTAGACTCAGCATAACAG-3′) and *Nkx2-5*-L-arm-R (5′-XhoI site-CAGGTTTCACAGCGCCAGGTG-3′) as well as *Nkx2-5*-R-arm-F (5′-EcoRI site-GATAAAAAAGGTAAGGAGAAC-3′) and *Nkx2-5*-R-arm-R (5′-EcoRV site-GGCAGGGTGGGCTACACAAGG-3′), respectively. The right-arm EcoRI-EcoRV fragment was cloned into pL453 as *Frt-Neo*^*r*^*-Frt-*R arm, and the left-arm XbaI-XhoI and *IRES-LacZ* XhoI-BamHI fragments were then simultaneously introduced to yield L arm-*IRES-LacZ-Frt-Neo*^*r*^*-Frt*-R arm. A XhoI-BglII fragment of mouse *Sox17* cDNA obtained by PCR with the primers 5′-XhoI site-GTCGCCACCATGAGCAGCCCGGATGCGGGA-3′ and 5′-TCTGCGTTGTGCAGATCTGGG-3′ and a BglII-XbaI fragment of the *Sox17* cDNA were simultaneously cloned into the plasmid to yield L arm-*Sox17-IRES-LacZ-Frt-Neo*^*r*^*-FRT-*R arm. EL250 cells transformed with the RP24-324A10 BAC clone were subjected to electroporation with the L arm-*Sox17-IRES-LacZ-Frt-Neo*^*r*^*-FRT-*R arm fragment and then to selection with kanamycin. Following removal of the *Neo*^*r*^ cassette by arabinose treatment (Flp induction), the BAC *Tg* was prepared and used for microinjection.

### RNA isolation, RT, and real-time PCR analysis

Embryonic hearts were dissected in ice-cold phosphate-buffered saline (PBS). Total RNA was isolated with the use of RNeasy Mini Kit (Qiagen) and was subjected to reverse transcription (RT) with the use of Superscript III (ThermoFisher Scientific) and an oligo(dT) primer. The resulting cDNA was subjected to real-time PCR analysis with specific primer sets (Table S4) and with the use of a QuantiTect SYBR Green PCR Kit and Rotorgene (Qiagen). Data were normalized by *Gapdh* expression.

### Histology

Mouse embryos were dissected in ice-cold PBS and fixed with 4% paraformaldehyde in PBS at 4 °C for 2 h. For assay of BrdU incorporation, pregnant mice were injected intraperitoneally with 500 µg of BrdU (Sigma-Aldrich) at 3 h before embryo removal. Fixed embryos were embedded in OCT compound (Sakura Finetek), frozen, sectioned at a thickness of 8 µm, and stained with primary and secondary antibodies (Table S5) as previously described^5,23^. DNA was counterstained with DAPI (Merck). Immunofluorescence micrographs were acquired with an LSM510 confocal (Zeiss) or BZ8000 fluorescence (Keyence) microscope. Whole mount *in situ* hybridization was performed as described^5,23^.

### Single-cell microarray analysis

The dissected hearts were treated with trypsin for 3 min at 37 °C to isolate single cells. First-strand cDNA was synthesized for 10 min at 50 °C with Superscript III reverse transcriptase (ThermoFisher Scientific). The cell type for each single-cell cDNA preparation was identified by PCR of marker gene expression with specific primer sets (Table S4). The T3 promoter was added to the 5′ end of each cDNA by PCR with the T3V3 primer (5′-CCAAGCTCGAAATTAACCCTCACTAAAGGGAGAATATCTCGAGGGCGCGCCGGATCC-3′) and V1dT_24_ primer (5′-ATATGGATCCGGCGCGCCGTCGACTTTTTTTTTTTTTTTTTTTTTTTT-3′). Cells were isolated from the heart ventricle of control (*Sox17*^fl/fl^) or *Sox17* knockout (*Mesp1*^Cre/+^/*Sox1*^fl/fl^) embryos at E8.5, and single-cell cDNA was synthesized by RT as previously described with some modifications^23^. The T3 promoter was added to the 5′ end of each cDNA by PCR. Virtual mRNAs were synthesized from the T3 promoter-cDNA constructs with a MEGAScript T3 Transcription Kit (ThermoFisher Scientific), and they (50 ng) were then labeled with Cy3 with the use of a Low RNA Input QuickAmp (One Color) Labeling Kit before hybridization with a SurePrint G3 Mouse GE v2 8 × 60 K Microarray with the use of a Gene Expression Hybridization Kit (Agilent). The processed data were analyzed statistically with Genespring GX software (Agilent). Expression levels of <50 were set to 50, per chip normalization was based on the 50th percentile, and per gene normalization was based on the median. Gene subtraction was performed as shown in Table S1. Gene lists were analyzed with Ingenuity Pathway Analysis software (Qiagen Bioinformatics). The microarray data obtained for this study are deposited in the Gene Expression Omnibus database (GEO, http://www.ncbi.nlm.nih.gov/geo) under the accession number GSE125323.

## Supplementary information

Supplementary Information

Gene List 1_up in KO EC_E8.5

Gene List 2_down in KO EC_E8.5

Gene List 3_up in KO MC_E8.5

Gene List 4_down in KO MC_E8.5
