# Supplementary material for: Endocardium differentiation through Sox17 expression in endocardium precursor cells regulates heart development in mice
Source: Sci Rep. 2019 Aug 16;9:11953. doi: 10.1038/s41598-019-48321-y (PMC6697751; doi:10.1038/s41598-019-48321-y)
Supplement: Supplementary file 1 — Supplementary Information [file 41598_2019_48321_MOESM1_ESM.pdf]

Supplementary Information

Endocardium differentiation through *Sox17* expression in endocardium precursor cells regulates heart development in mice

Rie Saba[1,2], Keiko Kitajima[3], Lucille Rainbow[4], Silvia Engert[5], Mami Uemura[6,7], Hidekazu Ishida[1,8], Ioannis Kokkinopoulos[1], Yasunori Shintani[1,9], Shigeru Miyagawa[10], Yoshiakira Kanai[6], Masami Kanai-Azuma[7], Peter Koopman[11], Chikara Meno[3], John Kenny[4,12], Heiko Lickert[5], Yumiko Saga[13], Ken Suzuki[1], Yoshiki Sawa[10], and Kenta Yashiro[1,2,10,14,*]

[1] Centres for Microvascular Research and for Endocrinology, William Harvey Research Institute, Barts and The London School of Medicine and Dentistry, Queen Mary University of London, London, UK

[2] Cardiac Regeneration and Therapeutics, Graduate School of Medicine, Osaka University, Osaka, Japan

[3] Department of Developmental Biology, Graduate School of Medical Sciences, Kyushu University, Japan

[4] Centre for Genomic Research, Institute of Integrative Biology, University of Liverpool, Liverpool, UK

[5] Institute of Diabetes and Regeneration Research, Helmholtz Zentrum München, Neuherberg, Germany

[6] Department of Veterinary Anatomy, The University of Tokyo, Tokyo, Japan

[7] Department of Experimental Animal Models for Human Disease, Graduate School of Medical and Dental Sciences, Tokyo Medical and Dental University, Tokyo, Japan

[8] Department of Pediatrics, Graduate School of Medicine, Osaka University, Osaka, Japan

[9] Department of Biophysics and Biochemistry, Graduate School of Medicine, Osaka University, Osaka, Japan

[10] Department of Cardiovascular Surgery, Graduate School of Medicine, Osaka University, Osaka, Japan

[11] Division of Molecular Genetics and Development, Institute for Molecular Bioscience, The University of Queensland, Brisbane, Australia

[12] Teagasc Food Research Centre, Moorepark, Co Cork, Ireland

[13] Division of Mammalian Development, Genetic Strains Research Center, National Institute of Genetics, Shizuoka, Japan

[14] Division of Anatomy and Developmental Biology, Department of Anatomy, Kyoto Prefectural University of Medicine, Kyoto Japan

* To whom correspondence may be addressed. Kenta Yashiro, MD, PhD, Department of Anatomy, Division of Anatomy and Developmental Biology, Kyoto Prefectural University of Medicine, 465 Kajii-cho, Kawaramachi-Hirokouji, Kamigyo-ku, Kyoto 602-8566, Japan. Tel: +81-75-251-5303. Email: kyashiro@koto.kpu-m.ac.jp

Supplementary Data Inventory

Supplementary Tables

Table S1. Microarray data and subtraction procedures.

Table S2. Pathway analysis for differentially expressed genes in endocardium cells of *Mesp1*$^{Cre/+}$/*Sox17*$^{fl/fl}$ versus *Mesp1*$^{+/+}$/*Sox17*$^{fl/fl}$ embryos at E8.5.

Table S3. Pathway analysis for differentially expressed genes in cardiomyocytes of *Mesp1*$^{Cre/+}$/*Sox17*$^{fl/fl}$ versus *Mesp1*$^{+/+}$/*Sox17*$^{fl/fl}$ embryos at E8.5.

Table S4. Primer sequences for RT-PCR.

Table S5. Antibodies for immunofluorescence.

Supplementary Figures

Figure S1. Co-expression of SOX17 and NKX2-5 in a mouse embryo at the EHF stage (E7.5).

Figure S2. SOX17 expression in the mesoderm of the mouse heart field.

Figure S3. Distribution of SOX17+ cells in the heart field of mouse embryos at the early somite stage.

Figure S4. Expression of arterial endothelial and endocardial marker genes in CPCs.

Figure S5. Expression of cardiac marker and *SoxF* genes in CPCs.

Figure S6. Mild to moderate gain-of-function phenotype for *Sox17* in mouse embryos.

Figure S7. Defective vascular remodeling in the yolk sac of mesoderm-specific *Sox17* mutant embryos at E9.5.

Figure S8. Anomalous looping of the heart tube in mesoderm-specific *Sox17* mutant embryos.

Figure S9. Cardiac defects associated with mesoderm-specific loss of function for *Sox17* in mouse embryos.

Figure S10. Reduced proliferation of cells in the endocardium and myocardium of mesoderm-specific *Sox17* mutant embryos.

Supplementary Data Set

Gene List 1. 96 genes from 114 probes up-regulated in *Sox17* knockout endocardial cell.

Gene List 2. 141 genes from 171 probes down-regulated in *Sox17* knockout endocardial cell.

Gene List 3. 64 genes from 85 probes up-regulated in *Sox17* knockout cardiomyocyte.

Gene List 4. 236 genes from 274 probes down-regulated in *Sox17* knockout cardiomyocyte.

Table S1. Microarray data and subtraction procedures.

Subtraction procedure	Endocardium E8.5 (nine-somite)	Myocardium E8.5 (nine-somite)
All probe sets	55821	55821
a) Flags detected in at least one condition	10496	7485
b) Expression level of >50 in at least one condition	5329	4515
c) $P < 0.05$ (*t* test) for WT vs. KO	460	712
d) Fivefold change for WT vs. KO	285	358
e) d/a (%)	2.71	4.78
Increased in KO	114	84
Decreased in KO	171	274

Probe sets whose expression in endocardium or myocardium cells of embryos at E8.5 (nine-somite stage) was affected by knockout (KO) of *Sox17* in mesoderm were extracted by filtering steps: a) "present" either in $Mesp1^{+/+}/Sox17^{fl/fl}$ (WT) or $Mesp1^{Cre/+}/Sox17^{fl/fl}$ (KO) embryos; b) a raw expression value of >50; c) significant difference between WT and KO by Student's *t* test ($P < 0.05$); d) fivefold difference in normalized signals between WT and KO; e) calculation of d/a ratio.

Table S2. Pathway analysis for differentially expressed genes in endocardium cells of *Mesp1*$^{Cre/+}$/*Sox17*$^{fl/fl}$ versus *Mesp1*$^{+/+}$/*Sox17*$^{fl/fl}$ embryos at E8.5.

Molecular and cellular functions	*P* value	No. of molecules
Cellular growth and proliferation	1.29E-02 – 3.29E-08	90
Cellular development	1.31E-02 – 3.52E-06	65
Cell cycle	1.24E-02 – 8.97E-06	35
DNA replication, recombination, and repair	9.65E-03 – 2.94E-05	25
Gene expression	1.32E-02 – 3.30E-05	53

Enriched pathways in the category of "Molecular and cellular functions" were determined by Ingenuity Pathway Analysis for the 285 probe sets in *Supplementary Data set*, Gene Lists 1 and 2.

Table S3. Pathway analysis for differentially expressed genes in cardiomyocytes of *Mesp1*$^{Cre/+}$/*Sox17*$^{fl/fl}$ versus *Mesp1*$^{+/+}$/*Sox17*$^{fl/fl}$ embryos at E8.5.

Molecular and cellular functions	*P* value	No. of molecules
Cell cycle	2.27E-02 – 2.24E-09	43
Cellular development	2.27E-02 – 2.24E-09	69
RNA posttranscriptional modification	2.27E-02 – 5.04E-07	19
Cellular growth and proliferation	2.27E-02 – 9.13E-05	95
Cellular assembly and organization	2.27E-02 – 1.12E-04	17

Enriched pathways in the category of "Molecular and cellular functions" were determined by Ingenuity Pathway Analysis for the 358 probe sets in *Supplementary Data set,* Gene Lists 3 and 4.

Table S4. Primer sequences for RT-PCR.

Gene name	Sense primer (5'-3')	Antisense primer (5'-3')
Acta2	TGTGTTTCTGTTAGGTGAGAATCA	TGGACCTTCCTCTGTTGAAGT
Actn2	CAGGTCATCGCCTCCTTCCGGATT	GTCGCTTTCCCCGTAGAGGGCAGA
Cryptic	CGCCAGAGGATCAAGAGAAT	CTTAGGAGCCTCAGCCCTTA
Dll4	AGCTGTGACTCCTGCCTCCAACCCC	CACATGAGCCCAGAGGCACCAGGAC
Etv2	AATAGCCGCGAGTTCCAG	CATAATTCATTCCCGGCTTC
Gapdh	ACAGTCCATGCCATCACTGCCACCC	CACAGCCTTGGCAGCACCAGTGGAT
Hey1	GGAGAAAGGTGTCTGTGCCC	ACTGTCTTGCTTGCTGCCAA
Hey2	TGTCTGTTTTGGGGAGAGGG	CATTCATTGTGATTTGGGATCAA
Kdr	GAGATTACAAGGCTTTCAGCA	CCTCCGTTTGAGATGAGAGAG
Nfatc1	GCTTTGTTGCATTCTCAAACA	GTCACCCACAGCACAGACA
Nkx2-5	TTGACGTAGCCTGGTGTCTC	TAGTGTGGAATCCGTCGAAA
Notch1	TCTGCTGCCCACCAAGTCCCTTTCC	TTAGGCATGGCACAGACACTGCCCC
Nrg1	GTATCAGCCATGACCCCCGGCTC	GCTGACTGCCACAGAGGGCATGGAC
Pecam1	CGGTTTCCTAAGGTCTGAGC	AGGCGAGGAGGGTTAGGTAT
Sox17	TTCTGTACACTTTAATGAGGCTGTTC	TTGTGGGAAGTGGGA TCAAG
Sox18	CTGTCGGCCTCCTCATTTACA	CAGGGCCTGAGGTTCCTA
Sox2	CATGAGAGCAAGTACTGGCAAG	CCAACGATATCAACCTGCATGG
Sox7	CACCCTGTGACACCCAAAGA	TACACGTGTCCAAGGGCAGA
Tal1	TTTTTGGTGGGTCTAGCCGTACTT	ACAGTACGACACTGACGCAAAGTG
Tbx5	CGTCGTGGAATTCAGAGTTG	CGATAGGTGCTGAGGAGTGA

Table S5. Antibodies for immunofluorescence.

Primary Antibodies	Target protein	Immune Animal	Company	Product No.	Dilution
Rb mAb to GFP	GFP	Rabbit	abcam	ab32146	1:1000
Anti-Sox17	Sox17	Goat	R&D	AF1924	1:100
Goat anti-cTNT	cardiac Troponin T	Goat	Hytest	4T19/2	1:3000
Rat anti-CD31 (MEC 13.3)	CD31	Rat	B&D Bioscience	550274	1:500
Ms mAb to MHC (MF20)	Myosin Heavy Chain	Mouse	Novus Biologicals	MAB4470	1:1000
Cleaved Caspase-3 (D175) Rabbit Ab	Cleaved Caspase-3	Rabbit	CST	9661S	1:100
Notch1 (D1E11) XP(R) Rabbit mAb	Notch1	Rabbit	CST	3608S	1:100
Rb pAb to Ki67	Ki67	Rabbit	abcam	ab15580	1:100
Phospho-Histone H3 (Ser10) antibody	p-H3	Rabbit	CST	9708	1:100
Rb pAB Nkx2-5	Nkx2-5	Rabbit	abcam	ab35842	1:100
Histone H3 (D1H2) XP(R) Rabbit mAb	Histone H3	Rabbit	CST	4499S	1:100
Mouse anti-BrdU (BU33)	BrdU	Mouse	Sigma	BU33	1:1000
**Secondary Antibodies**	**Target protein**	**Immune Animal**	**Company**	**Product No.**	**Dilution**
Donkey anti-rabbit IgG Alexa 488	rabbit IgG	Donkey	Jackson ImmunoResearch Laboratories	711-546-152	1:1000
Donkey anti-rat IgG Alexa 594	rat IgG	Donkey	Jackson ImmunoResearch Laboratories	712-586-153	1:1000
Donkey anti-goat IgG Alexa 488	goat IgG	Donkey	Jackson ImmunoResearch Laboratories	705-546-147	1:1000
Donkey anti-mouse IgG Alexa 594	mouse IgG	Donkey	Jackson ImmunoResearch Laboratories	715-586-151	1:1000
Donkey anti-mouse IgG Alexa 488	mouse IgG	Donkey	Jackson ImmunoResearch Laboratories	715-546-151	1:1000

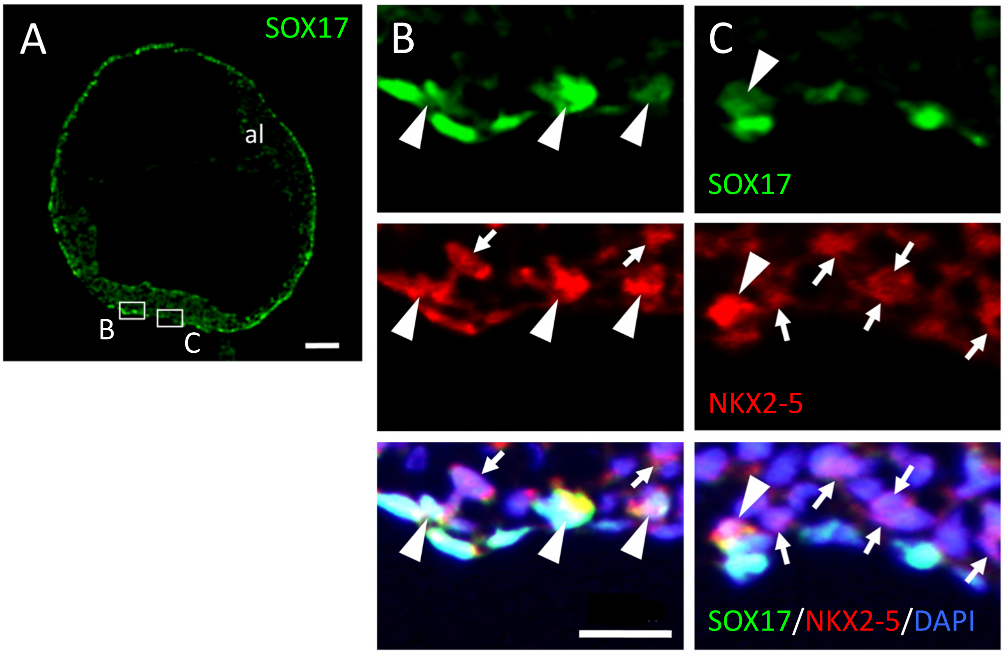

Fig. S1. Co-expression of SOX17 and NKX2-5 in a mouse embryo at the EHF stage (E7.5). Expression of SOX17 (green) and NKX2-5 (red) was examined by immunofluorescence analysis. Nuclei (blue) were stained with DAPI. The boxed regions in *A* are shown at higher magnification in *B* and *C*. Arrows and arrowheads indicate NKX2-5 single-positive cells and cells positive for both SOX17 and NKX2-5, respectively. Al, allantois. Scale bars, 100 μm (*A*) and 50 μm (*B* and *C*).

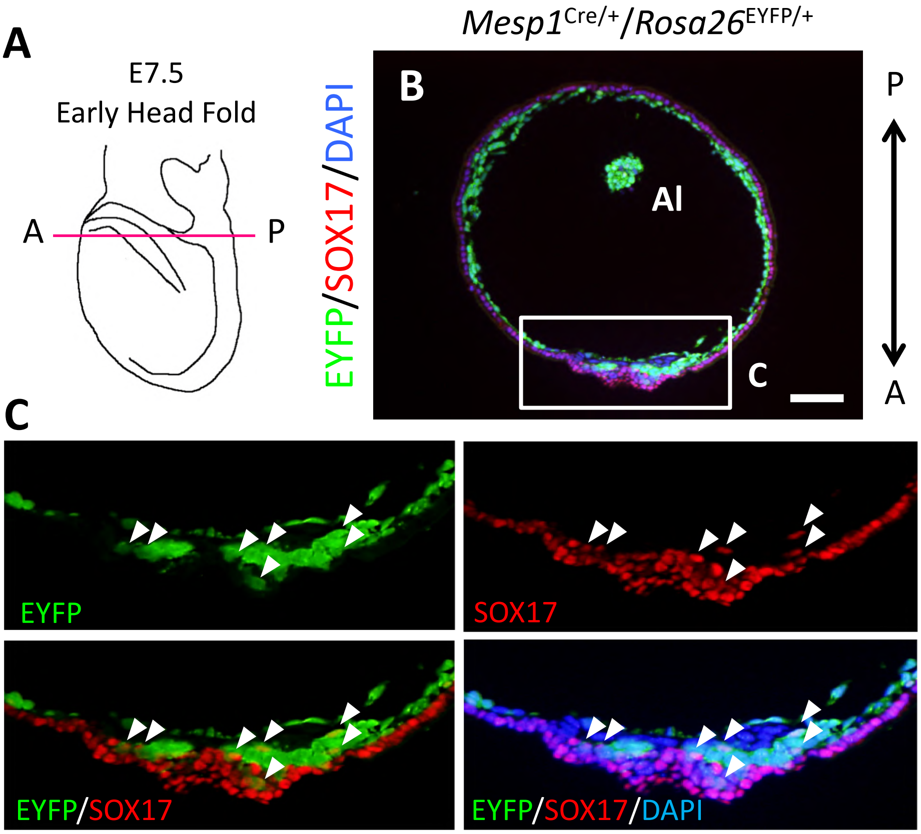

Fig. S2. SOX17 expression in the mesoderm of the mouse heart field. (*A*) Schematic representation of a mouse embryo at the early head fold stage (E7.5) as a left lateral view. The magenta line shows the sectional plane along the anterior (A)–posterior (P) axis in *B*. (*B* and *C*) Immunofluorescence micrographs of SOX17+ cells (red) in a *Mesp1*$^{\mathrm{Cre/+}}$/*Rosa26*$^{\mathrm{EYFP/+}}$ mouse embryo. EYFP (green) marks mesoderm cells, and nuclei (blue) were stained with 4′,6-diamidino-2-phenylindole (DAPI). The boxed region in *B* is shown at higher magnification in *C*. Arrowheads indicate EYFP+, SOX17+ cells. Al, allantoic bud. Scale bar, 100 μm.

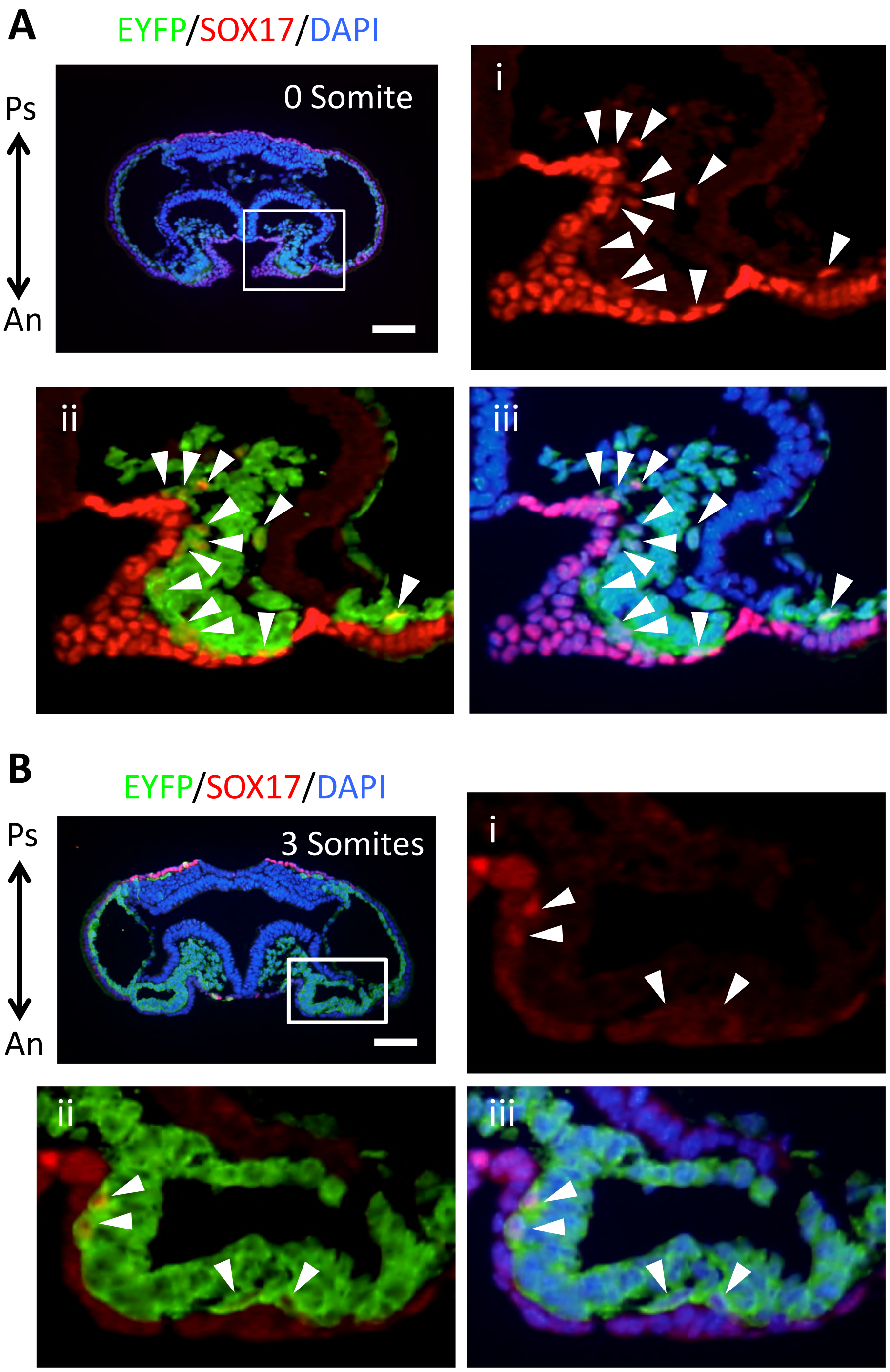

Fig. S3. Distribution of SOX17+ cells in the heart field of mouse embryos at the early somite stage. Expression of SOX17 (red) and EYFP (green) in the heart field of *Mesp1*$^{\mathrm{Cre/+}}$/*Rosa26*$^{\mathrm{EYFP/+}}$ mouse embryos at the zero-somite (*A*) and three-somite (*B*) stages was examined by immunofluorescence analysis. Nuclei (blue) were stained with DAPI. The boxed region in the upper left panel for each embryo is shown at higher magnification in the corresponding panels labeled i to iii. Arrowheads indicate cells positive for both SOX17 and EYFP. An, anterior; Ps, posterior. Scale bars, 100 μm.

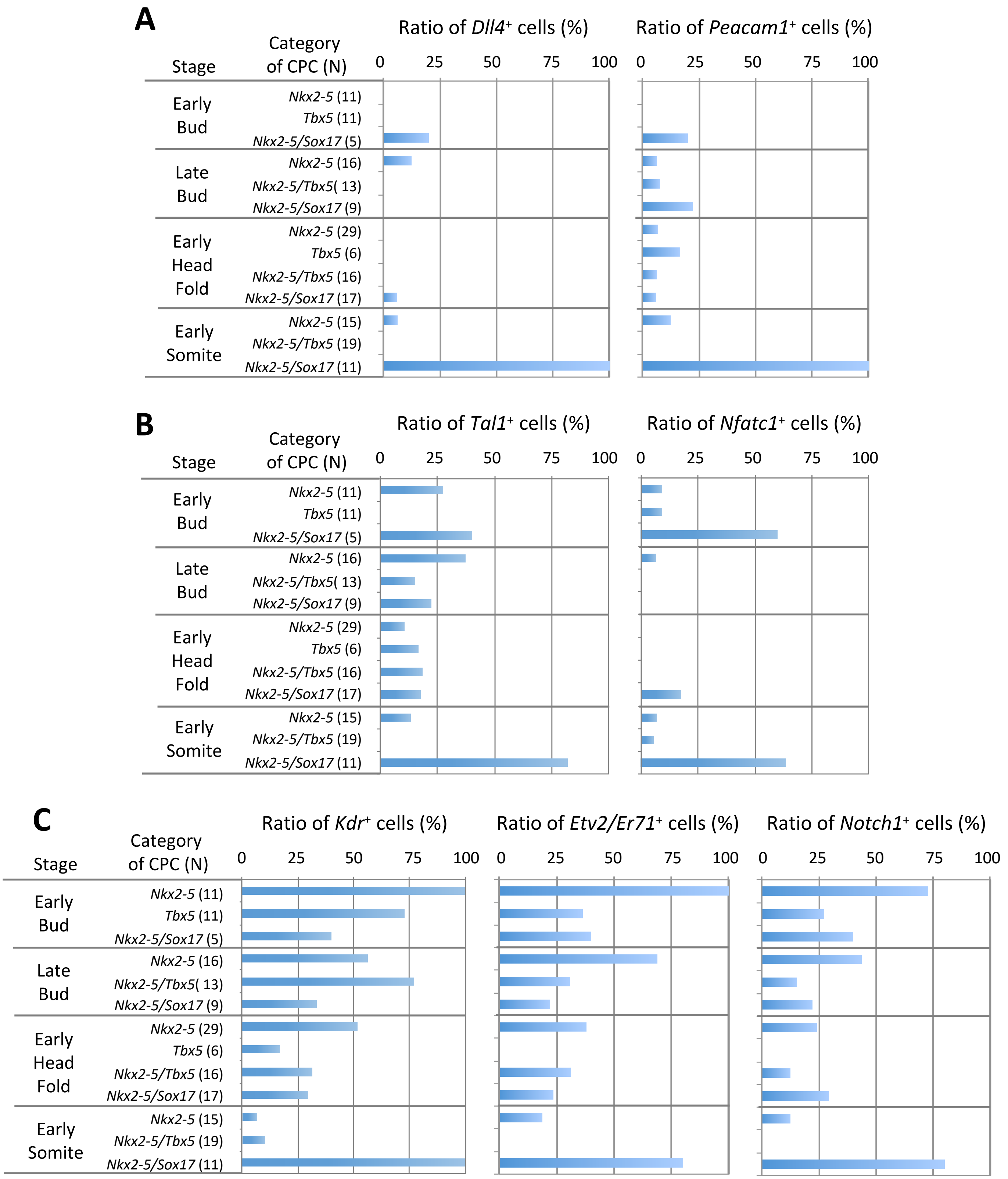

Fig. S4. Expression of arterial endothelial and endocardial marker genes in CPCs. The ratio of *Dll4*+ or *Pecam1*+ cells (*A*), *Tal1*+ or *Nfatc1*+ cells (*B*), or *Kdr*+, *Etv2*/*Er71*+ or *Notch1*+ cells (*C*) among various categories of CPCs in mouse embryos at the indicated stages was determined by PCR analysis of single-cell cDNA. The number of cells analyzed is shown in parentheses.

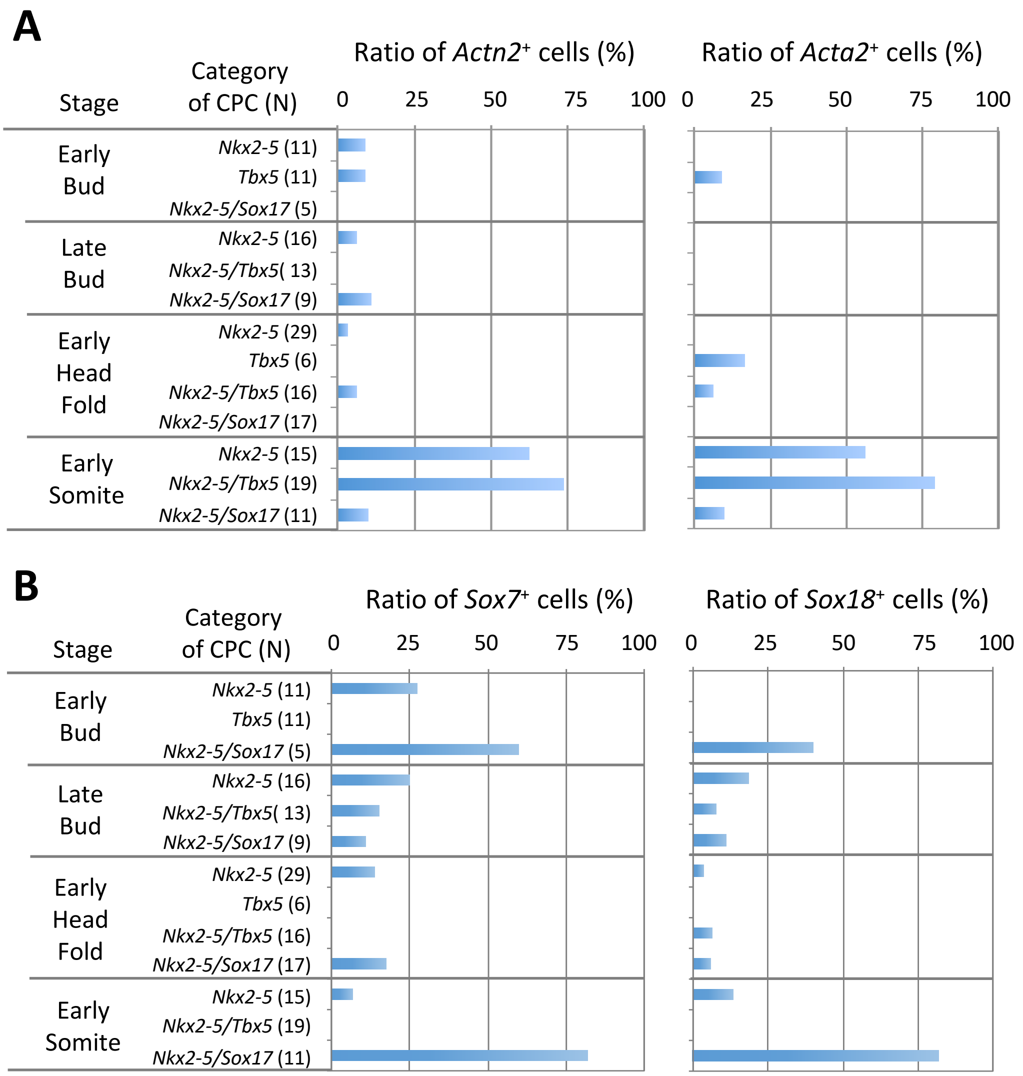

Fig. S5. Expression of cardiac marker and *SoxF* genes in CPCs. The ratio of *Actn2*+ or *Acta2*+ cells (*A*) or of *Sox7*+ or *Sox18*+ cells (*B*) among various categories of CPCs in mouse embryos at the indicated stages was determined by PCR analysis of single-cell cDNA. The number of cells analyzed is shown in parentheses.

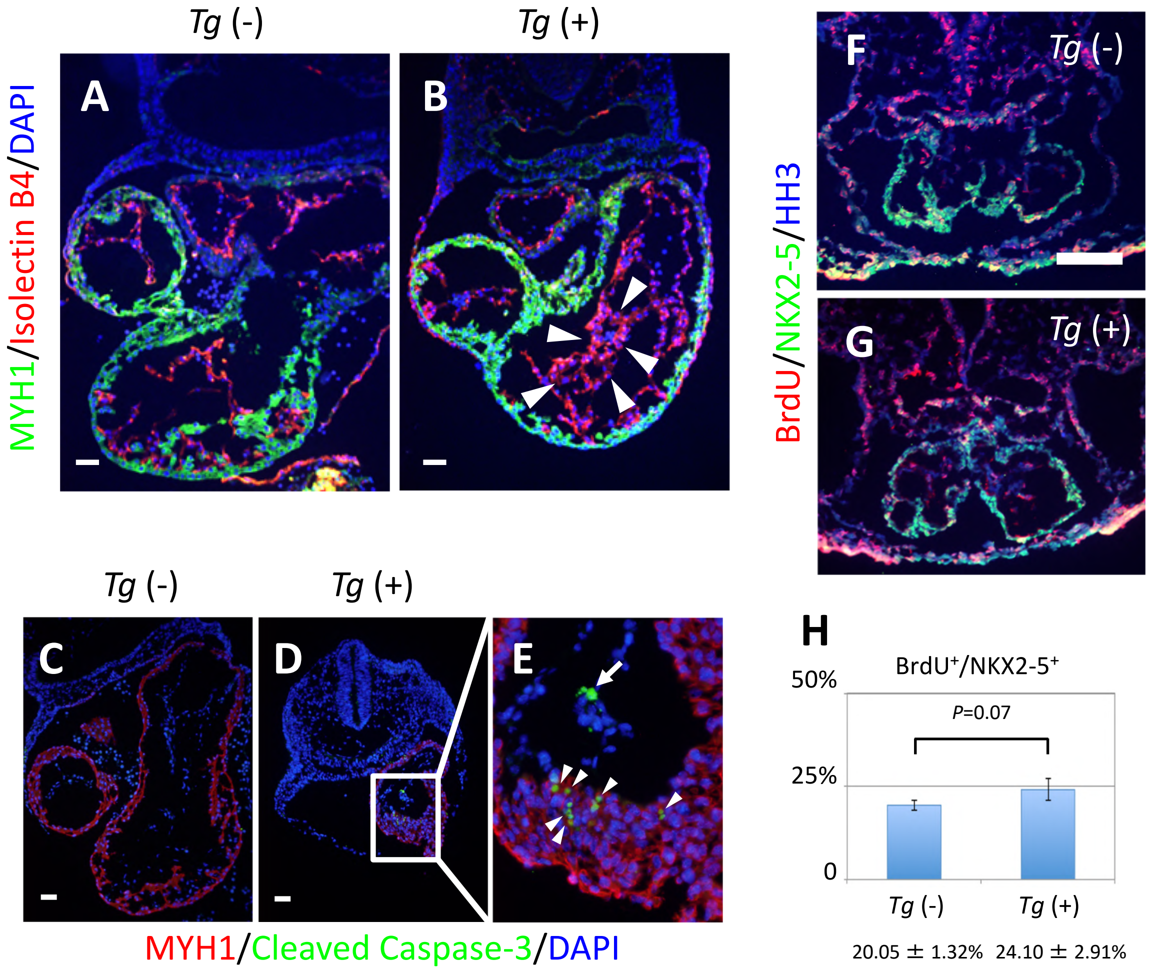

Fig. S6. Mild to moderate gain-of-function phenotype for *Sox17* in mouse embryos. (*A* and *B*) Fluorescence micrographs for immunostaining of MYH1 (cardiomyocytes, green and isolectin B4 staining (endocardium, red) in the heart of WT (*A*) and BAC *Nkx2-5*$^{\text{Sox17-IRES-LacZ-BghA}}$ transgenic (*B*) embryos at E9.5. Nuclei (blue) were stained with DAPI. Arrowheads indicate aggregated endocardium cells. Scale bars, 20 μm. (*C–E*) Immunofluorescence micrographs for MYH1 (red) and cleaved caspase-3 (apoptotic cells, green) in *Tg*(–) (*C*) and *Tg*(+) (*D*) embryos at E9.5. Nuclei (blue) were stained with DAPI. The boxed region in *D* is shown at higher magnification in *E*. The arrow and arrowheads indicate apoptotic cells positive for cleaved caspase-3 in the endocardium and myocardium, respectively Scale bar, 100 μm. (*F* and *G*) Immunofluorescence micrographs for bromodeoxyuridine (BrdU) incorporation (red) as well as for NKX2-5 (green) and histone H3 (HH3, nucleus, blue) in *Tg*(–) (*F*) and *Tg*(+) (*G*) embryos at E8.. Scale bar, 100 μm. (*H*) Proportions of BrdU+ cells among NKX2-5+ cells in the heart of *Tg*(–) and *Tg*(+) embryos at E8.5. Data are means ± SD (*N* = 3 embryos). The *P* value was determined by Student's *t* test.

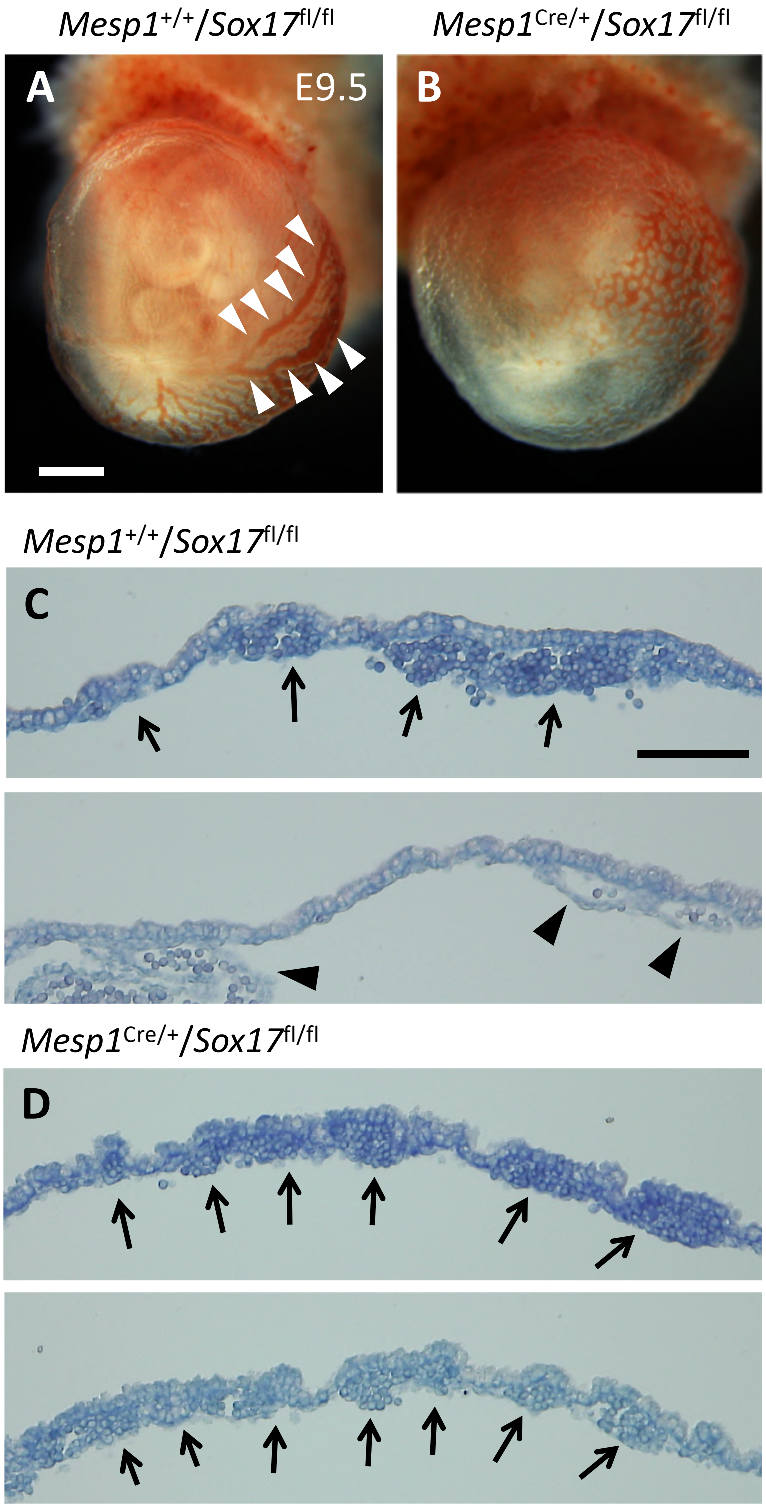

Fig. S7. Defective vascular remodeling in the yolk sac of mesoderm-specific *Sox17* mutant embryos at E9.5. (*A* and *B*) Macroscopic observation of the yolk sac of *Mesp1*$^{+/+}$/*Sox17*$^{fl/fl}$ (*A*) and *Mesp1*$^{Cre/+}$/*Sox17*$^{fl/fl}$ (*B*) embryos. Remodeled large blood vessels similar to those indicated by the arrowheads in *A* are not apparent in the mutant. Scale bar, 500 μm. (*C* and *D*) Giemsa staining of the yolk sac of WT (*C*) and mutant (*D*) embryos. Blood islands (arrows) containing primary erythrocytes are formed in both WT and mutant embryos, whereas remodeled blood vessels (arrowheads) are present only in the WT embryo. Upper and lower panels show different areas of the yolk sac. Scale bar, 100 μm.

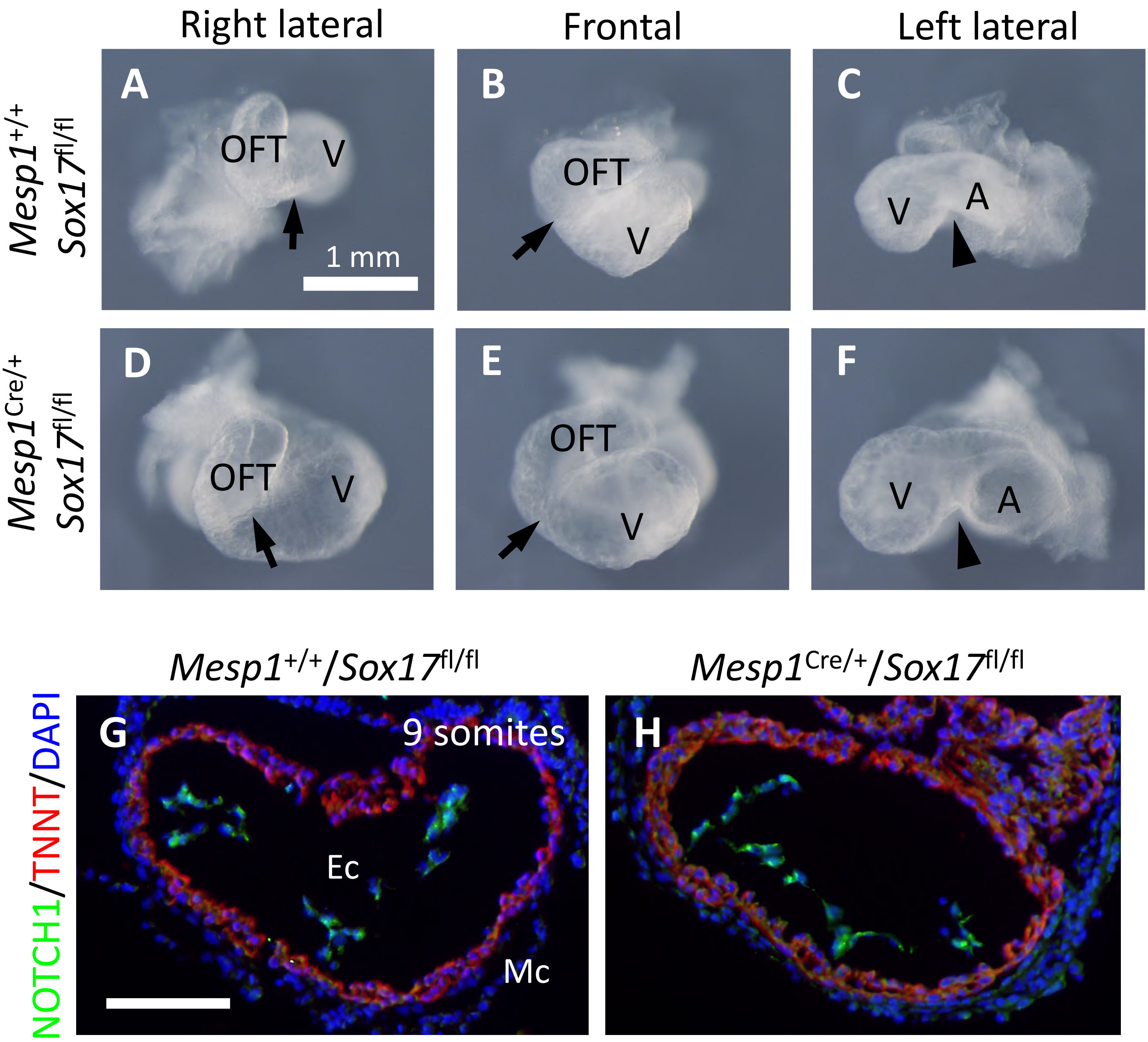

Fig. S8. Anomalous looping of the heart tube in mesoderm-specific *Sox17* mutant embryos. (*A–F*) The heart of *Mesp1*$^{+/+}$/*Sox17*$^{fl/fl}$ (*A–C*) or *Mesp1*$^{Cre/+/}$*Sox17*$^{fl/fl}$ (*D–F*) embryos at the 22-somite stage (E9.5) is shown in right lateral (*A* and *D*), frontal (*B* and *E*), and left lateral (*C* and *F*) views. Arrows and arrowheads indicate the borders of the outflow tract (OFT) and ventricle (V) and of the ventricle and atrium (A), respectively. (*G* and *H*) Immunofluorescence micrographs for NOTCH1 (green) and TNNT (red) in the heart of *Mesp1*$^{+/+}$/*Sox17*$^{fl/fl}$ (*G*) and *Mesp1*$^{Cre/+/}$*Sox17*$^{fl/f}$ (*H*) embryos at the nine-somite stage (E8.5). Nuclei (blue) were stained with DAPI. Ec, endocardium; Mc, myocardium. Scale bar, 100 μm.

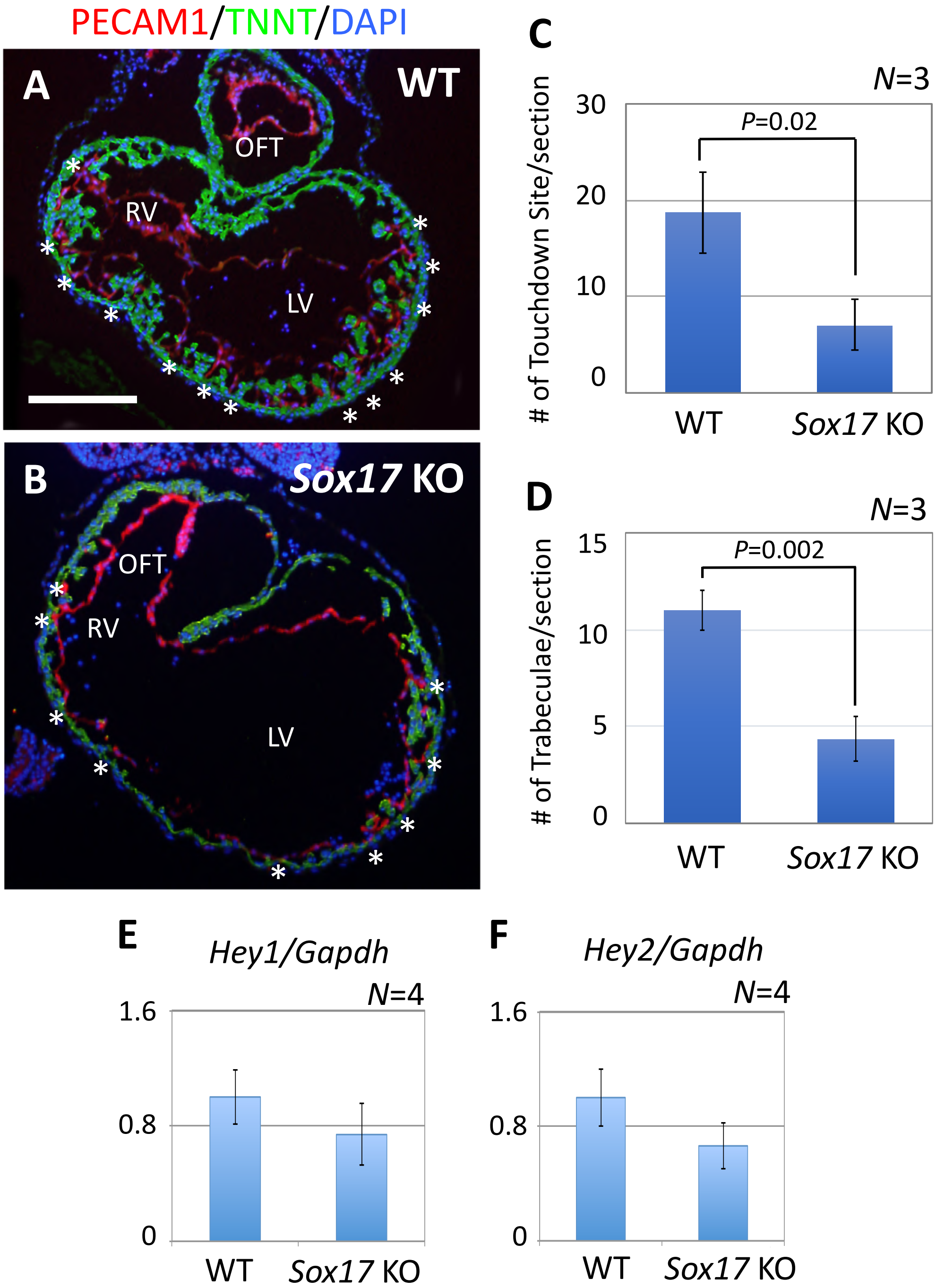

Figure S9. Cardiac defects associated with mesoderm-specific loss of function for *Sox17* in mouse embryos. (*A*, *B*) Immunofluorescence micrographs in the heart of *Mesp1*$^{+/+}$/*Sox17*$^{fl/fl}$ (WT, *A*) and *Mesp1*$^{Cre/+}$/*Sox17*$^{fl/fl}$ (*Sox17* KO, *B*) embryos at E9.5. Scale bar, 100 µm. Red, PECAM1; Green, TNNT; Blue, DAPI. Asterisks indicate the touch down sites of the endocardium to the myocardium. (*C*, *D*) Averaged numbers of touchdown sites (*C*) and trabeculae (*D*), which are composed of more than three cardiomyocytes, per section in the ventricle of WT and *Sox17* KO at E9.5. *P* values (Student's *t* test) are indicated. Means ± SD. (*E*, *F*) Reverse transcription and real-time PCR analysis of the relative expression levels for the *Hey1* (*E*) and *Hey2* (*F*) in the heart of WT and *Sox17* KO embryos at E9.5.

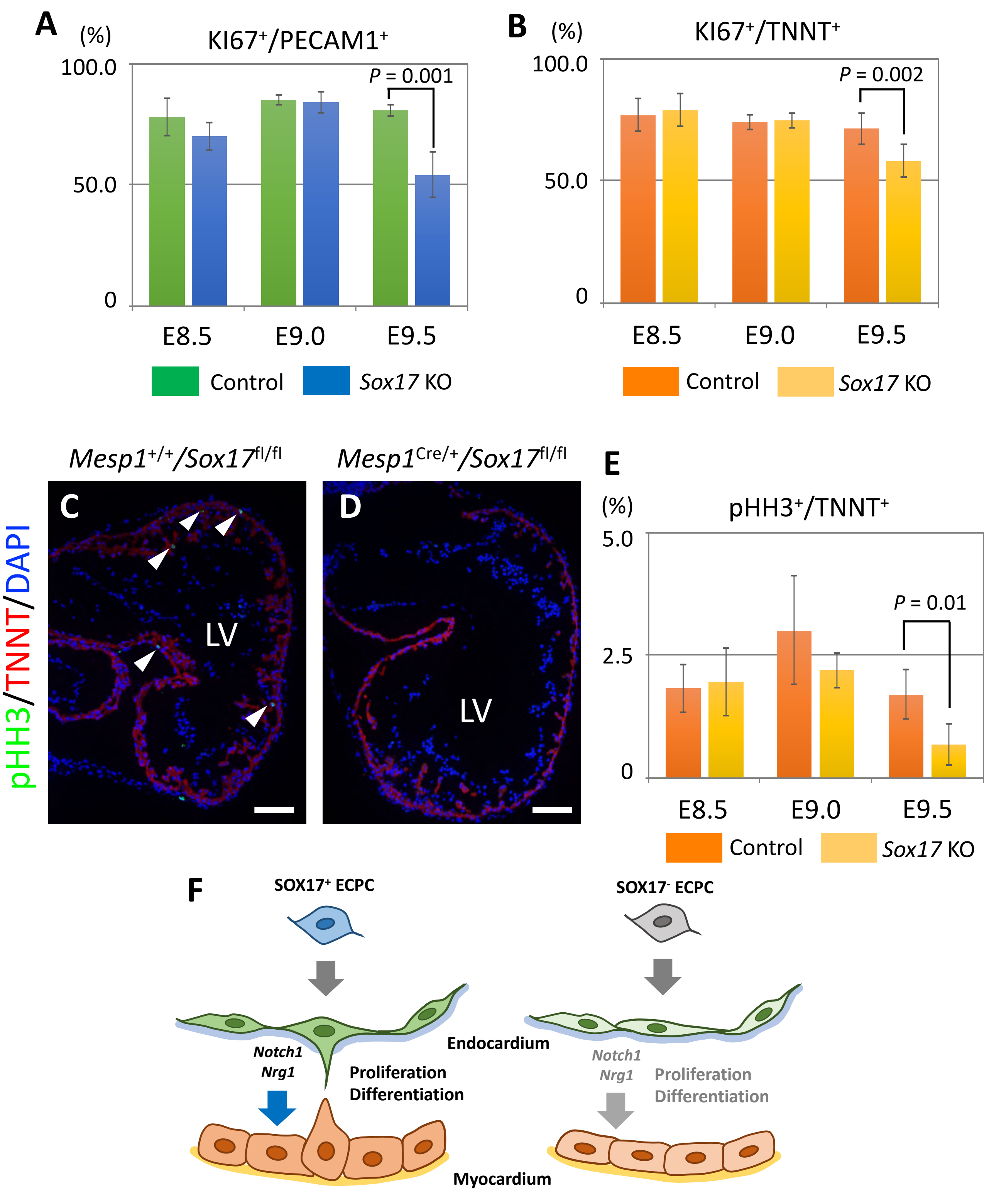

Fig. S10. Reduced proliferation of cells in the endocardium and myocardium of mesoderm-specific *Sox17* mutant embryos. (*A* and *B*) Proportions of KI67+ cells among PECAM1+ endocardium cells (*A*) or TNNT+ cardiomyocytes (*B*) of *Mesp1*$^{+/+/Sox17\text{fl/fl}}$ (control) and *Mesp1*$^{\text{Cre}/+/}$*Sox17*$^{\text{fl/fl}}$ (*Sox17* KO) embryos from E8.5 to E9.5. Data are means SD ($N = 4$ embryos). Only significant P values calculated by Student's t test are shown. (*C* and *D*) Immunofluorescence micrographs of pHH3 (green) and TNNT (red) in the myocardium of the left ventricle (LV) of *Mesp1*$^{+/+}$/*Sox17*$^{\text{fl/fl}}$ and *Mesp1*$^{\text{Cre}/+/}$*Sox17*$^{\text{fl/fl}}$ embryos at E9.5. Nuclei (blue) were stained with DAPI. Arrowheads indicate pHH3-positive cells. Scale bars, 50 μm. (*E*) Proportion of pHH3+ cells among TNNT+ cardiomyocytes in mutant and control embryos from E8.5 to E9.5. Data are means SD ($N = 4$ embryos). Only significant P values calculated by Student's t test are shown. (*F*) Model for the role of *Sox17* in endocardium development and function. ECPC, endocardium precursor cell.
